# Flexible processing of distractor stimuli under stress

**DOI:** 10.1038/s41598-024-61162-8

**Published:** 2024-05-11

**Authors:** Imke M. Duehnen, Susanne Vogel, Nina Alexander, Markus Muehlhan, Andreas Löw, Thomas Jacobsen, Mike Wendt

**Affiliations:** 1 Experimental Psychology Unit, Faculty of Humanities and Social Sciences, Helmut Schmidt University/University of the Federal Armed Forces Hamburg, Hamburg, Germany; 2 Department of Psychology, Faculty of Human Science, Medical School Hamburg, Hamburg, Germany; 3 ICAN Institute for Cognitive and Affective Neuroscience, Medical School Hamburg, Hamburg, Germany; 4 Department of Psychiatry and Psychotherapy, University of Marburg, Marburg, Germany; 5 Center for Mind, Brain and Behavior, University of Marburg, Marburg, Germany

**Keywords:** Psychology, Human behaviour

## Abstract

Acute stress is assumed to affect executive processing of stimulus information, although extant studies have yielded heterogeneous findings. The temporal flanker task, in which a target stimulus is preceded by a distractor of varying utility, offers a means of investigating various components involved in the adjustment of information processing and conflict control. Both behavioral and EEG data obtained with this task suggest stronger distractor-related response activation in conditions associated with higher predictivity of the distractor for the upcoming target. In two experiments we investigated distractor-related processing and conflict control after inducing acute stress (Trier Social Stress Test). Although the stressed groups did not differ significantly from unstressed control groups concerning behavioral markers of attentional adjustment (i.e., Proportion Congruent Effect), or event-related sensory components in the EEG (i.e., posterior P1 and N1), the lateralized readiness potential demonstrated reduced activation evoked by (predictive) distractor information under stress. Our results suggest flexible adjustment of attention under stress but hint at decreased usage of nominally irrelevant stimulus information for biasing response selection.

## Introduction

Stress is ubiquitous in most people’s life and, intuitively, most individuals believe that stress generally impairs cognitive functioning. However, despite an increasing number of publications concerning the effects of stress on attention, executive functions, and memory, the precise effects of acute stress on information processing are still not fully understood. Following a classic account of the effect of acute stress on information processing, referred to as *Easterbrook’s hypothesis*^1^, exposure to stress leads to a withdrawal of cognitive resources from the processing of irrelevant stimuli, thus enhancing selective attention. This conjecture has been investigated by analyzing interference effects, evoked by distractor stimuli, in conflict tasks like the Stroop task^2^ (for an overview, see^3^) under stress. Although various studies indeed demonstrated reduced Stroop interference in stressed participants^4–6^, it has been argued that these results might reflect a strategic change in task processing unrelated to selective attention, such as a general reduction in response threshold to speed up task completion, leaving less time for distractor processing to affect responding^7^.

Conflict tasks, such as the famous Stroop tasks^2^, typically allow an evaluation of the degree of selective attention by contrasting performance in conditions in which a distractor stimulus is associated with a different response than the target stimulus (i.e., incongruent condition) with a condition in which both target and distractor are associated with the same response (i.e., congruent condition). The performance difference between these conditions is denoted the *congruency effect*. Intriguingly, the size of the congruency effect varies with the proportions of congruent and incongruent trials. It is typically larger when the Proportion Congruent (PC) is increased. This pattern of results is denoted as the *Proportion Congruent Effect* (*PCE*, for overviews see^8,9^), and has been observed in various conflict tasks, such as the Stroop task^10–12^, the Eriksen Flanker task^13,14^, and response priming tasks (in which the presentation of a distractor stimulus precedes the presentation of the target^15–19^). The PCE has been assumed to result from the interplay of monitoring and regulation processes in the service of performance optimization, thus representing a paradigm case of cognitive (or executive) control. The most prominent account of the PCE, referred to as Conflict Monitoring Theory^20^, posits that the attentional bias favouring target-related over distractor-related stimulus information is continuously adjusted to the degree of response conflict (i.e., simultaneous activation of multiple responses). Consequently, the impact of distractor information is assumed to be optimized based on previous utility (i.e., increased or decreased after little vs. much conflict, respectively).

Research concerning effects of acute stress on executive control has yielded a range of subtle findings which do not easily combine to a coherent picture and do not seem easily explained in terms of a single mechanism such as withdrawal of attention from irrelevant stimulus information, however. As an example, Husa, Buchanan, and Kirchhoff^21^ investigated acute stress effects on the use of different processing strategies (i.e., proactive vs. reactive cognitive control) in an AX-Continuous Performance Test in which a cue and a probe (i.e., imperative stimulus) letter are presented successively with varying degree of predictability of the former regarding the latter. The authors observed no evidence for a difference in processing strategies between the stress group and the control group. Within the stress group, however, participants reporting a higher level of subjective stress less frequently appeared to use the proactive control strategy and more frequently appeared to use the reactive control strategy, suggesting difficulty in maintaining the task-related goals prior to presentation of the target.

Proactive control has been extensively investigated by analysing effects of varying the length of a preparation interval preceding a task switch trial (for an overview of task switching, see e.g., Kiesel et al.^22^). Both Steinhauser et al.^23^ and Plessow et al.^24^ applied a stress manipulation to a task switching protocol and observed an increase in task switch costs. However, whereas in the study of Steinhauser et al.^23^, acute stress resulted in an increase in task switch reaction time (RT) costs selectively in conditions of a long preparation interval, the study of Plessow et al.^23^ demonstrated enhanced task switch costs for stressed participants only in error rates but regardless of the length of the preparation interval. Although suggestive of a deficit in cognitive flexibility under stress, such discrepancies in experimental results preclude identifying the precise changes in processing strategies.

Regarding attentional adjustment to PC, Booth and Sharma^7^ assessed the impact of acute stress on the Stroop effect, manipulating the PC between different blocks of experimental trials. The authors observed a significant PCE in a control condition involving a moderate level of white noise but not in a stress condition involving loud white noise (i.e., 65 dBC and 84 dBC, respectively), although only for a subgroup of participants equipped with high working memory (WM) capacity. Reasoning that an increase in selective attention would lower the likelihood of noticing a change in PC, the authors interpreted their finding as evidence for Easterbrook’s hypothesis.

Recent developments in the domain of conflict-related attentional adjustment provide a novel option to investigate the cognitive processes underlying the PCE in more detail. In particular, the task procedure used in the current study—referred to as the Temporal Flanker Task^25^ (for a detailed description see methods of Experiment 1)—has previously yielded a number of behavioural and physiological results potentially useful for the detailed investigation of stress-elicited alterations in executive functioning. In the Temporal Flanker Task, two stimulus items (i.e., target and distractor) are presented successively in each trial. Participants are asked to respond to the second stimulus by pressing a corresponding response key and ignore the first item. Both stimuli are drawn from the same set of items (e.g., four different letters of the alphabet), and feature the same physical properties (i.e., color, size, location etc.) when used as target (i.e., second item of a trial) or as distractor (i.e., first item of a trial). Thus, temporal order of presentation is the only property that allows participants to discriminate the target from the distractor. Analogous to the Eriksen flanker task, trials can be categorized according to whether the same or different letters appear as target and distractor, constituting a congruent vs. an incongruent condition, respectively, and the size of the congruency effect (i.e., the difference in response performance in congruent and incongruent trials) is considered an indicator of the degree of attentional focusing on the target. The Temporal Flanker Task has been used successfully to investigate attentional adjustment to conflict conditions. In particular, various studies yielded a PCE comparable to more traditional conflict tasks mentioned above (i.e., the Stroop task and the Eriksen flanker task)^15–19^.

A striking advantage of presenting the distractor and the target successively relates to the possibility of analyzing physiological responses to the distractor before target onset, allowing assessment of distractor-evoked physiological effects uncontaminated by target processing. Applying EEG recording, at least three studies observed more pronounced brain potentials evoked by the distractor in blocks of trials associated with high PC than in blocks of trials associated with low PC, demonstrating enhanced processing under conditions of higher distractor utility (i.e., when most trials were congruent)^15,17,19^. Moreover, manipulations of the stimulus onset asynchrony (SOA) provided insights into proactive control applied to the processing distractor information. Specifically, increasing the SOA is typically associated with a reduction (and sometimes even reversal) of the congruency effect^26,27^. This reduction has been attributed to progressive inhibition of distractor processing^26–28^. Combining the SOA manipulation with a PC manipulation, Gillich et al.^16^ observed additive effects on the congruency effect, consistent with the assumption that two independent control processes are applied in parallel, reducing the conflict effect when more time was available before the target occurred and, at the same time, adjusting the strength of distractor processing to its utility for response selection (i.e., assigning more attentional weight to the distractor under conditions of high PC, that is, when the majority of trials is congruent). In light of the above-mentioned findings suggesting stress-related impairment in proactive control, the aim of the current study was to examine the impact of acute stress on distractor processing in high and low PC conditions.

In sum, the Temporal Flanker Task offers various options of identifying stress-related changes in information processing extending a general increase in selective attention, such as impairments of conflict monitoring, attentional adjustment, or regulation of distractor-evoked response activation. In the current study, we report two experiments comparing performance in the Temporal Flanker Task after participating in the Trier Social Stress Test (TSST)^29^ vs. a non-stressful control condition. In Experiment 1, we collected only behavioral data, exerting careful experimental control to rule out a non-attentional alternative account of the PCE. In Experiment 2, we added electrophysiological recording to obtain measures of distractor processing uncontaminated by target information (i.e., during the SOA interval).

## Experiment 1

Accounting for a PCE in terms of attentional adjustment requires ruling out an alternative interpretation often referred to as contingency learning. This alternative interpretation arises from a confounding of PC condition and distractor-specific contingencies. For illustration, consider the case of a Stroop task, in which the two color-words RED and GREEN are presented in the two colors red and green. When the PC is high (e.g., 75%) each of the two words will occur together with the corresponding response (i.e., the response associated with the color named by the word) in 75% of the cases. By contrast, when the PC is low (e.g., 25%), each word will occur together with the response associated with the other color in 75% of the trials. These item-specific (i.e., distractor-related) contingencies might facilitate responding in congruent trials, when the PC is high, and in incongruent trials, when the PC is low, based on associative learning rather than attentional adjustment. Deconfounding of PC and distractor-specific contingencies has been achieved by dividing the set of stimuli into two distinct subsets, referred to as inducer items and diagnostic items, respectively, and manipulating PC only for the former, such that an effect of PC on diagnostic items cannot be explained by associative learning^8,10,16,30^. Applying this method in Experiment 1, we expected to observe a PCE in in both inducer and diagnostic items. Assuming a stress related deficit in attentional adjustment or change in cognitive control strategy, we further expected a reduced PCE in the group of participants that was exposed to the TSST.

### Methods Experiment 1

#### Participants

We based the sample size on previous studies that demonstrated an influence of acute stress induced by the TSST on effects assumed to reflect executive control. For instance, the studies of Steinhauser et al.^23^ or of Plessow et al.^24^ involved 40 and 48 participants, respectively.

Fourty-eight students of the Medical School Hamburg participated in the study (24 females, 24 males, mean age 23.02, range = 19–30, 44 right-handed). They received partial course credit in exchange for participation. All participants had normal or corrected-to-normal vision according to self-report. The study was conducted in accordance with the Declaration of Helsinki with ethics approval obtained from the institutional review board of the Medical School Hamburg (MSH-2017/26). All procedures were carried out with written informed consent of the participants. Participants were pseudo randomly assigned to the stress or control group, considering equal gender ratio and PC condition of the first block.

Using a telephone screening, we excluded individuals with current medication or illicit drug intake, or any past or current medical condition potentially affecting stress reactivity, e.g., thyroid dysfunction, thyroid or allergy medication. To avoid creating a highly selective sample within this age group, smokers and women using oral contraceptives were not excluded. Importantly however, the groups did not differ in smoking status or use of contraception [*X*^2^(1, N = 47) = 0.36, *p* = 0.55, and *X*^2^(1, N = 47) = 1.61, *p* = 0.20, respectively]. Women who were not using hormonal contraceptives were tested during the luteal phase of their cycle as the female cycle can affect cortisol release^31^.

All testing took place between 1 and 7 p.m. Participants were instructed not to eat, drink caffeinated beverages, or engage in excessive sports activities one hour prior to testing.

#### Apparatus, stimuli, task and procedure

The paradigm was presented on a Lenovo PC, Intel (R) Pentium (R) CPU G3220 at 3.00 GHz, Ram: 4.00 GB; 22″-LCD-Display screen. Responses were detected by a Serial Response Box, Model 200a (Psychology Software Tools, Inc.). Stimulus presentation and recording of response times were controlled by E-Prime 2.0 (Psychology Software Tools, Inc.) under Windows 7 Professional (64 Bit). For the experiment, participants were seated approximately 60 cm in front of the screen. For distractor and target stimulus, the letters A, B, C, and D were presented in white color on a gray rectangle (1.6 × 1.6 cm) in the center of the screen. Letters extended from 1.4 cm vertically and 1.2 cm horizontally, subtending approximately 1.3° of visual angle vertically and 1.1° of visual angle horizontally. Responses were mapped in alphabetical order to the response keys from the left to right. Participants pressed the response keys with the index and middle fingers of their left and right hand.

**Figure 1:**
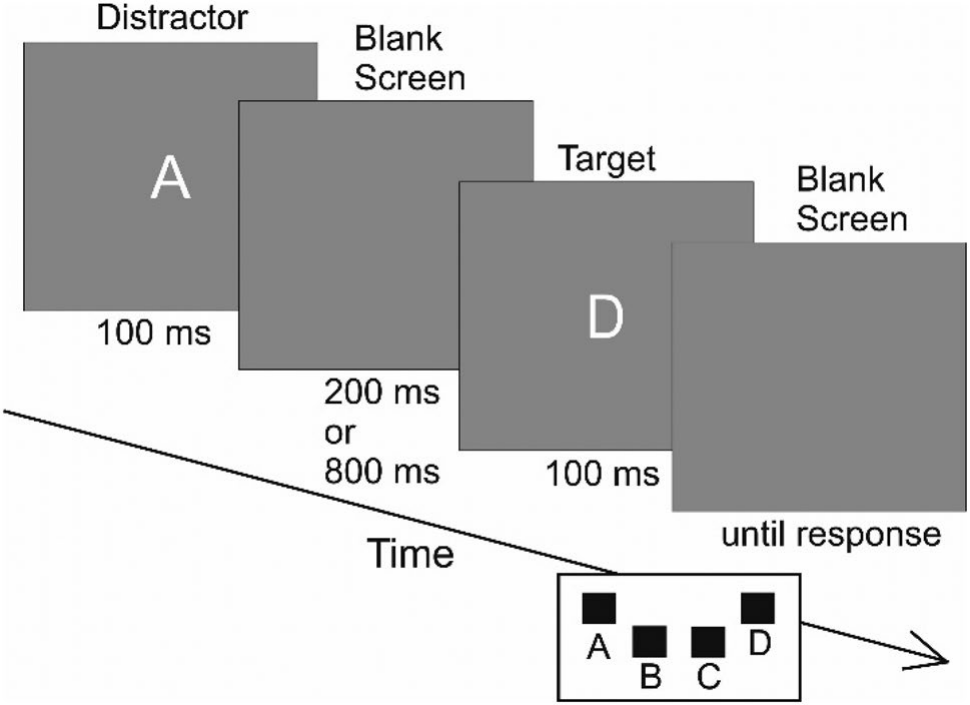
Temporal structure of an experimental trial. Note that distractor presentation time (i.e., 100 ms) and the time interval of the blank screen following distractor presentation (i.e., 200 ms or 800 ms) amount to stimulus onset asynchronies of 300 ms and 900 ms, respectively.

An example trial from the Temporal flanker task used in Experiment 1 can be seen in Fig. 1. For each trial, a distractor was presented prior to the target with a stimulus onset asynchrony of either 300 ms (short SOA) or 900 ms (long SOA). Distractor (i.e., first letter presented in the trial) and target (i.e., second letter presented in the trial) were presented for 100 ms each. Participants were instructed to respond to the target with the corresponding key-press as fast and accurately as possible while ignoring the distractor. A trial was categorized as congruent if the same letter occurred as target and distractor, and as incongruent if the letters used as target and distractor differed. After each block, mean RT and accuracy were displayed until the participant continued with the next block. An erroneous response was indicated by presenting *falsch* (i.e., wrong) on the screen for 500 ms. After a correct response, a blank screen was presented for 500 ms. The distractor of the following trial was presented following an interval of another 500 ms in both cases.

Two 2 × 2 mappings of the stimuli were used, with the letters A and D as inducer items and the letters B and C as diagnostic items for half of the participants. For the other half of the participants the letters B and C served as inducer items and the letter A and D as diagnostic items. The experiment consisted of 8 blocks with 96 trials. From the 96 trials of each block 72 trials consisted of inducer items, and 24 trials as diagnostic items to control for confounding effects of associative learning^16^.

In the high-PC blocks, a high proportion of trials containing inducer items were congruent (83.33%), and the subset of diagnostic items was presented with a constant frequency of 50% congruent trials, resulting in an overall congruency of 75% in the high-PC blocks. Whereas in the low-PC blocks a low proportion of the trials with inducer items were congruent (50%), and the subset of diagnostic items was also presented with a frequency of 50% congruent trials. If the PCE that occurs in the inducer stimuli also transfers to the diagnostic stimuli, it is arguably an effect of cognitive control to the utility of the distractor rather than only an effect of associative learning (for a detailed explanation see^16^). Both the PC (high vs. low) of the first block and assignment of stimuli as either inducer or diagnostic stimuli (A and D vs. B and C) were counterbalanced over participants.

**Figure 2:**
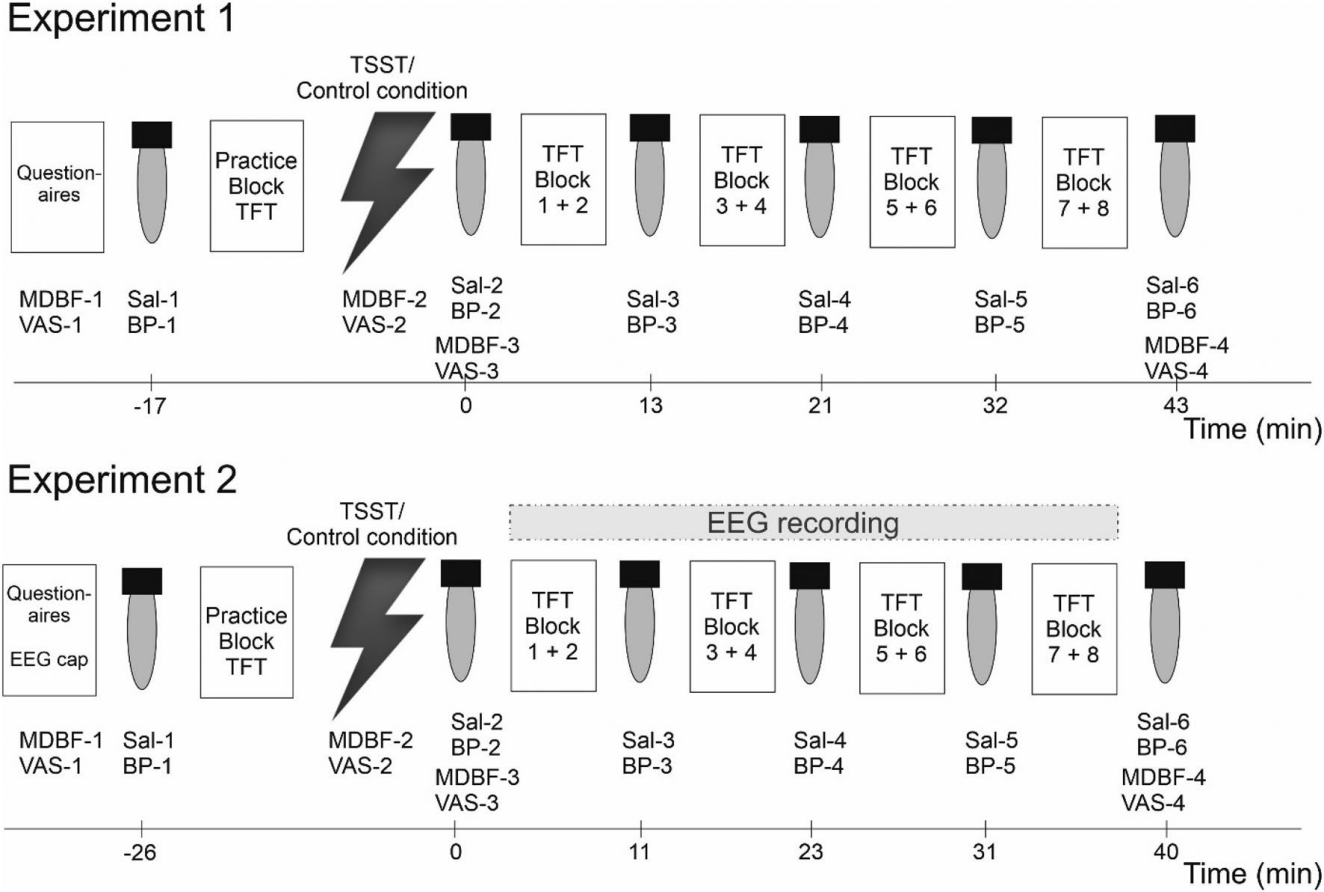
Time course of experiment 1 and 2. Note: BP = blood pressure and heart rate. MDBF = subjective mood questionnaire. Sal = salivary sample. TFT = temporal flanker task. TSST = Trier Social Stress Test or non-stressful control procedure. VAS = visual analogue scale. Time is averaged over all participants for both experiments.

The overall procedure of the experiment is depicted in Fig. 2. Before participants started with the temporal flanker task, they completed a German mood questionnaire (short form A of the German version of the Multidimensional Mood Questionaire (MDBF^32^), visual analogue scales (VAS, assessing perceived subjective anxiety, tension and distress), a questionnaire evaluating attentional control (Attentional Control Scale (ACS^33^), a questionnaire evaluating quality of sleep (Pittsburgh Sleep Quality Index (PSQI^34^), and the NEO – Five-Factor Inventory (NEO-FFI^35^). The ACS and the PSQI were not analyzed for this article. The MDBF assesses current low vs. elevated mood, restlessness vs. calmness, and sleepiness vs. wakefulness. Each score of the short form of the MDBF scales can range from 4 to 20, where high scores represent elevated mood, calmness, and wakefulness, respectively. The VAS consisted of three visual analogue scales to assess subjective anxiety, tension, and distress on three scales ranging from 1 (‘not at all’) to 9 (‘very much’). Moreover, participants’ vital signs were measured (blood pressure, heart rate) using an automatic wrist blood pressure monitor (Omron RS2, the Netherlands) and a baseline saliva sample was obtained (see Supplementary methods). Next, they performed a practice block of the temporal flanker task of 32 trials, where each distractor could appear with each target. Participants were then brought to a separate room for the Trier Social Stress Test (TSST^29^) or a non-stressful control procedure of equal duration, both of which also included the MDBF and VAS. After the TSST or the non-stressful control condition, the third MDBF and VAS, a blood pressure reading and saliva sample were obtained. Afterwards, participants executed the main paradigm with 8 blocks à 96 trials. After block 2, 4, 6 and 8, the investigator took saliva samples (sample 3 to 6) and blood pressure readings from the participant (see Fig. 2). Detailed Description of salivary cortisol assay can be found in the supplement.

At the end of the task participants completed the MDBF and VAS for a last time and were debriefed about the study procedures.

#### Stress induction protocol

The TSST is considered to be the gold-standard for mild laboratory stress induction in humans leading to robust subjective, endocrine, and autonomic stress responses by combining socio-evaluative threat and unpredictability^36^. It simulated a 15-min job interview and consisted of a 5-min preparation phase (including subjective mood assessment), a 5-min public speech about the participant’s eligibility for his/her favorite job, and a 5-min difficult mental-arithmetic task (counting backwards from 2043 in steps of 17). During the entire 15 min, participants were instructed that they were videotaped and evaluated by two neutral, non-reinforcing committee members in laboratory coats. In contrast, participants in the control condition spoke alone about a topic of their choice and performed an easy arithmetic task (counting forwards from 0 in steps of 10). They were neither videotaped nor evaluated. A similar control procedure has been used in other studies and did not result in increases in cortisol or negative mood^37^.

#### Data analysis

Due to a strong increase in salivary cortisol concentration following the control procedure, accompanied by pronounced increases in blood pressure and heart rate, one control participant was excluded from all analyses, and due to missing cortisol samples two additional participants were excluded, resulting in data from 45 participants (24, stress, 21 control).

To assure successful induction of acute stress, subjective and physiological data were analyzed using an ANOVA-type non-parametric test for treatment effects with global alternatives for repeated measures data in factorial designs (nparLD package v2.1^38^; RStudio, v1.1.463; Rstudio Team 2015) with the factors Group (stress, control) and time. A non-parametric test was used because the data was not normally distributed. To control for subjective stress effects on cognitive control we calculated correlations of the increase of values (i.e., timepoint 2 − timepoint 1) derived by the MDBF (elevated mood, calmness and wakefulness) and VAS (subjective anxiety, tension and distress), with the individual PCE data in the stress group using Spearman rho.

Subsequently, we calculated the area under the curve with respect to increase (AUCi)^39^ to obtain an integrative measure of cortisol reactivity. Spearman’s rho was then used to assess the association between increase of salivary cortisol concentration and cognitive control, as indicated by the PCE in RTs.

For the analysis of behavioral data from the Temporal Flanker Task, data from the first three trials of each block were considered warm-up trials and not included in the analyses (3.13%). For the analysis of RTs, only trials associated with a correct response (2.41% trials excluded) that fell within the range of 200–2500 ms were included (2.75% trials excluded). Additionally, trials following error trials were excluded from the analysis (2.13%).

ANOVAs with repeated measures on the factors Congruency (congruent, incongruent), PC (high PC, low PC), Stimulus Type (inducer, diagnostic), SOA (short, long) and the between subject factor Group (stress, control) were conducted on RTs and error rates (Supplementary Table S5 and S6) using the ez package (v4.0-0^40^) in RStudio (v1.1.463; RStudio Team 2015). For correlational analyses, the PCE was calculated by subtracting the congruency effect (CE) in the low PC condition from the congruency effect in the high PC condition (i.e., CE-high-PC – CE-low-PC).

## Results

### Subjective and physiological data

Our analyses on the MDBF revealed that stress did not significantly affect positive mood, calmness and wakefulness (Group × Time: all *p*s > 0.08, Supplementary Table S1).

However, stress had significant effects on all three scales of the VAS over time (Group × Time: subjective anxiety, F(1,2.72) = 7.27, *p* < 0.001; tension, F(1,2.58) = 9.47, *p* < 0.001; distress, F(1,2.56) = 11.63, *p* < 0.001, Supplementary Table S2). Using Mann–Whitney-U-Tests we observed that subjective anxiety, tension and distress did not differ between control and stress group at the beginning of the experiment (W = 313.5, *p* = 0.339; W = 273.5, *p* = 0.965; and W = 295, *p* = 0.688, respectively), but the stress group showed significantly more anxiety, tension and distress after the stress condition compared to the control group (W = 146.5, *p* = 0.005; W = 106, *p* < 0.001; and W = 126.5, *p* = 0.001, respectively).

**Figure 3:**
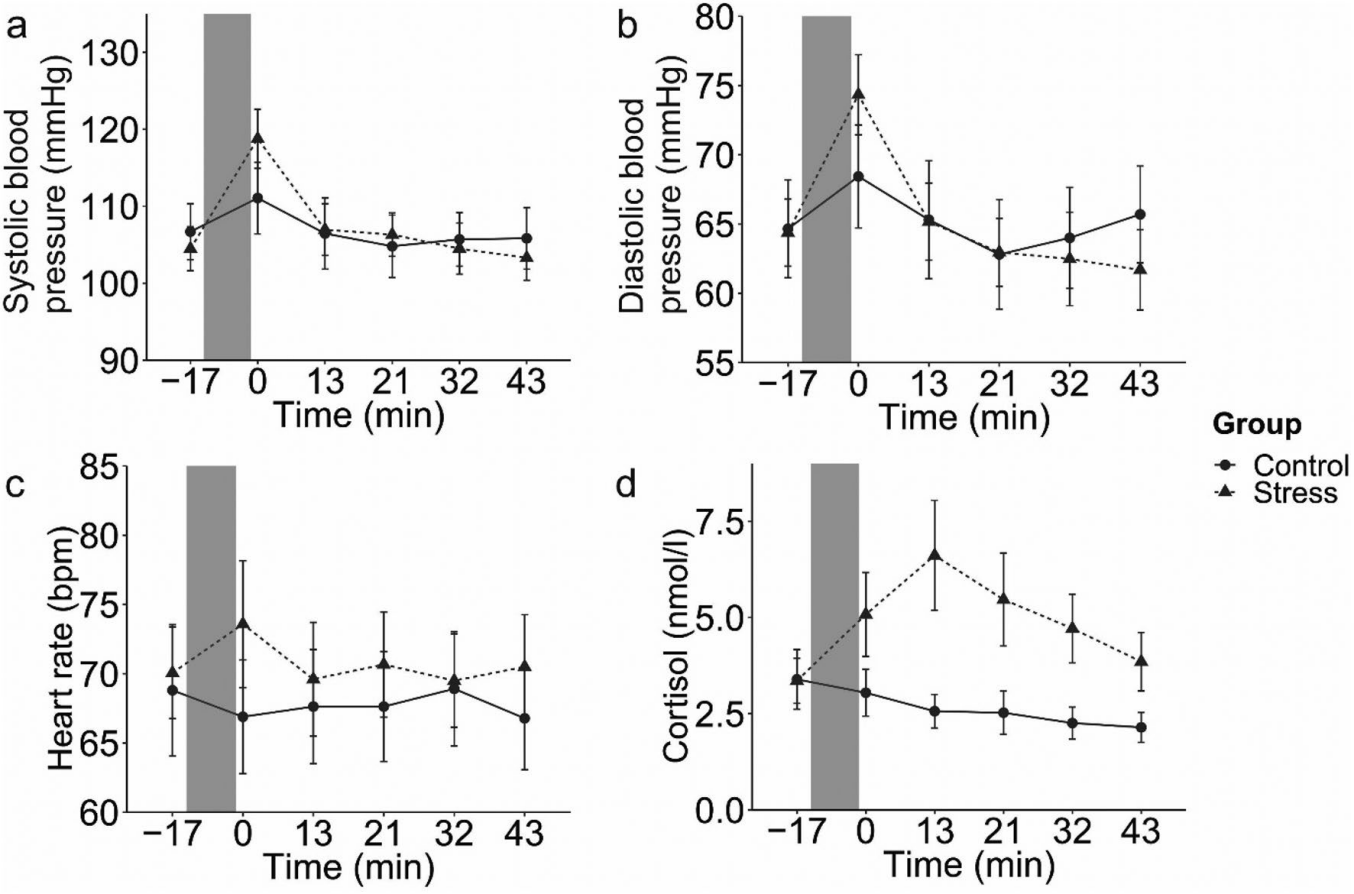
Systolic (**a**) and diastolic blood pressure (**b**), heart rate (**c**) and salivary cortisol concentration (**d**) for the stress and control group separately with mean data and error bars indicating 95% Confidence intervals. The gray bar indicates the stress/control manipulation.

Furthermore, successful stress induction was indicated by activations of the autonomic nervous system (ANS) and hypothalamus–pituitary–adrenal (HPA) axis. Systolic and diastolic blood pressure (Fig. 3a,b), increased in response to the TSST (Group × Time: F(1,4.32) = 4.63, *p* < 0.001, and F(1,4.18) = 4.66, *p* < 0.001, respectively), but heart rate (Fig. 3c) did not (F(1,4.04) = 2.20, *p* = 0.065). Post-hoc Mann–Whitney-U Test confirmed that systolic and diastolic blood pressure did not differ significantly between groups at the beginning of the experiment (W = 319.5, *p* = 0.360; and W = 285.5, *p* = 0.848, respectively) but immediately after stress/control condition (W = 150, *p* = 0.008; and W = 145, *p* = 0.005, respectively).

Finally, we observed a significant cortisol response in the stress group but not in the control group (Fig. 3d) (Group × Time: F(1,2.55) = 15.66, *p* < 0.001). Post-hoc Mann–Whitney-U Test revealed that cortisol concentration was not significantly different before the TSST (W = 270, *p* = 0.907) but increased after the TSST until the end of the experiment (W = 114, *p* = 0.001; W = 50.5, *p* =  < 0.001; W = 72, *p* =  < 0.001; W = 73.5, *p* =  < 0.001; W = 116, *p* = 0.001, for each timepoint respectively).

### Behavioral data (Temporal Flanker Task)

**Figure 4:**
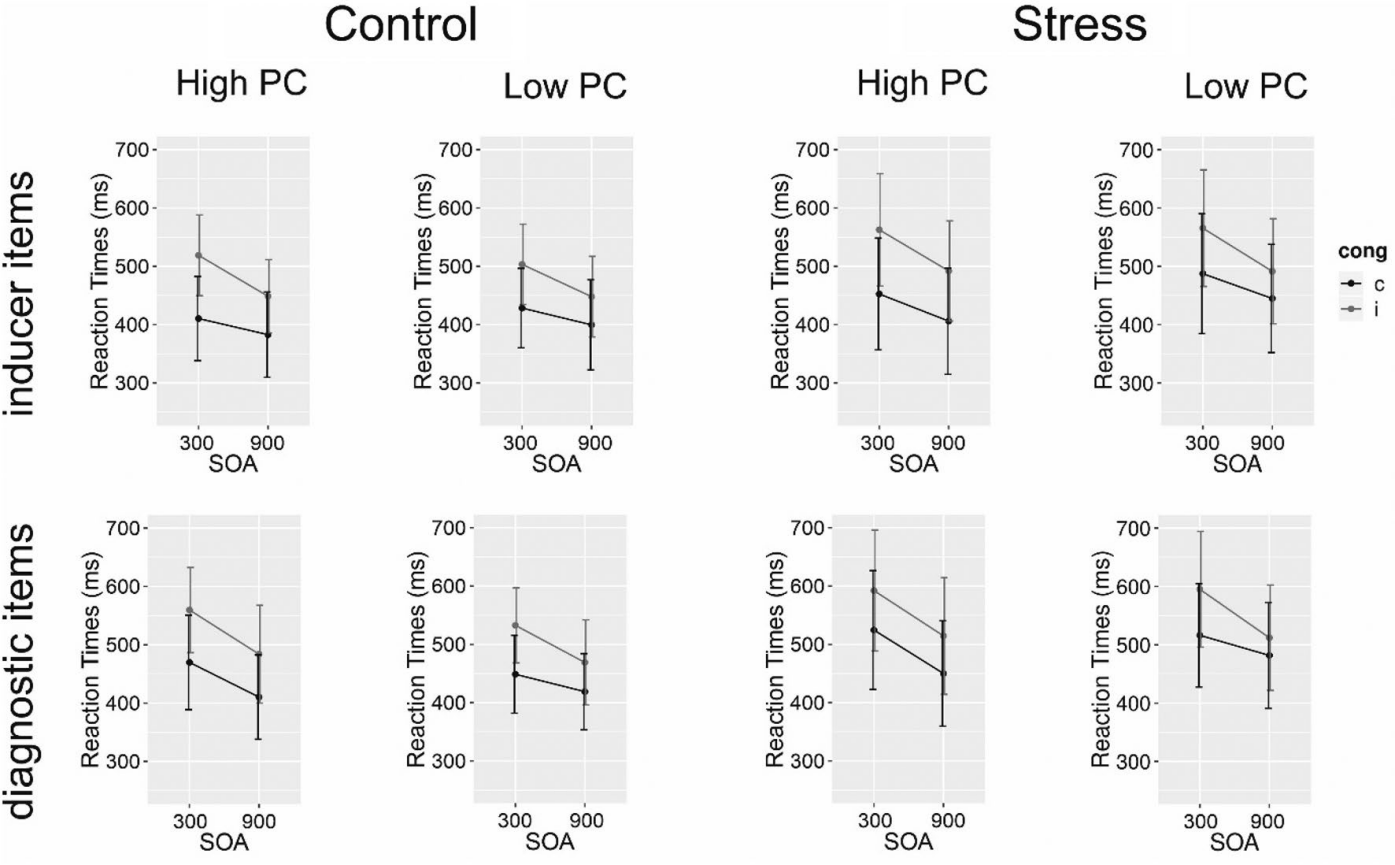
RTs for congruent (c) and incongruent (i) inducer (top) and diagnostic (bottom) stimuli from the control group and stress group, separately, with error bars indicating 95%- Confidence intervals.

*Reaction times* Mean RT data is displayed in Fig. 4. As expected from prior work^16^, repeated measures ANOVA for RTs (supplementary Table S5) yielded significant main effects for the factors Congruency, Stimulus Type, and SOA, demonstrating faster responding for congruent vs incongruent trials (432 ms and 514 ms, respectively), faster responding in trials associated with inducer items than in trials associated with diagnostic items (450 ms and 502 ms, respectively), and faster responding in trials with long SOA vs short SOA (437 ms and 488 ms, respectively).

Further replicating previous findings^16^, both interactions of Congruency × PC and Congruency × SOA were significant, reflecting a larger congruency effect for the high-PC condition (i.e., PCE) (CE in high-PC condition: 100 ms; CE in low PC condition: 63 ms), and a reduction of the congruency effect when SOA was increased (CE in short SOA trials: 97 ms; CE in long SOA trials: 66 ms). Furthermore, PC × SOA entered into a significant interaction, indicating decreased RTs in high-PC blocks compared to low-PC blocks after long SOA (422 ms and 452 ms, respectively), but no difference between PC blocks after short SOA (471 ms and 504 ms, for high-PC and low-PC blocks, respectively). Additionally, the two-way interaction PC × Stimulus Type reached significance, reflecting increased RT in low-PC vs. high-PC blocks for the inducer items (471 ms and 429 ms, respectively) and no difference for the diagnostic items between PC blocks (501 ms and 502 ms, for low-PC and high-PC blocks respectively).

The three-way interaction Congruency × PC × Stimulus Type reached significance, showing a reduced (but still significant) PCE for the diagnostic compared to the inducer stimuli (12 ms and 30 ms, respectively).

Most important to our research question, however, we observed no effect between the stress and the control group on RT (see Fig. 4; main effect and all interactions, ps > 0.100, mean RT for control group: 442 ms; mean RT for stress group: 480 ms).

The three-way interaction Group × Congruency × PC did not reach significance (F(1,45) = 0.33, *p* = 0.569), meaning we did not observe a reduction of the PCE under stress.

*Error rates* Mirroring RT results, a rm ANOVA for error rates (Supplementary Table S6, see Supplementary Tables S3 and S4 for descriptive data) revealed main effects for the factor SOA, and Stimulus Type, as well as a significant interaction of Congruency × PC, demonstrating increased error rates for short SOA trials (2.1% and 1.5% for short and long SOA trials respectively), increased error rates for diagnostic stimuli compared to inducer stimuli (2.7% and 1.5%, respectively), and a PCE (CE for high-PC blocks: -0.9; CE for low-PC blocks: < 0.01). Again, however, we found no main effect between both experimental groups on error rates (all *p*s > 0.07, mean error rates for control group: 1.8%; mean error rates for stress group: 1.8%).

To assess possible interindividual differences in stress reactivity, we then tested whether individual PCE values (in RTs) were correlated with the cortisol response in the stress group (area under the curve with respect to increase). However, we found no evidence for an effect of cortisol release on attentional adjustment (rho = −0.036, *p* = 0.869). Furthermore, we observed no correlation of the PCE with increase in subjective stress (all rho’s < 0.25, all *p*s > 0.19).

To control for possible effects of smoking or hormonal contraceptives, the ANOVAs on RT and error rates were repeated with the covariates smoking (yes/no) and use of hormonal contraceptives (yes/no). However, the results remained highly similar and importantly, again no effect of stress was detected.

### Bayesian analysis

Our conclusions are based on null hypothesis significance testing and therefore only allow to reject the null but not the alternative hypothesis. Thus, we repeated our analysis regarding stress effects on behavior with a Bayesian approach. The Bayes Factor was computed using the BayesFactor (v0.9.124.2^41^) package in RStudio. Default Cauchy priors were used (scaling factor r = 0.707) with 10.000 iterations. The Bayes Factor was then calculated by comparing the full model including the three-way interaction of Congruency, PC and Group (Congruency + Group + Congruency × Group + PC + Congruency × PC + Group × PC + Congruency × Group × PC) with the equivalent model excluding the three-way interaction and only including the two-way interactions of Congruency and PC, Congruency and Group, and PC and Group (Congruency + Group + Congruency × Group + PC + Congruency × PC + Group × PC).

For interpretation, a Bayes factor between 1 and 3 relates to anecdotal evidence, between 3 and 10 substantial evidence, between 10 and 30 strong evidence, between 30 and 100 very strong evidence, and over 100 decisive evidence for the tested hypothesis (Jeffreys, 1961).

For RT data, comparing the H0 (model including the three-way interaction of Congruency, PC and Group) with the H1 model including only the two-way interactions of Congruency and PC, Congruency and Group, and PC and Group), the Bayes Factor was found to be BF_10_ = 4.34*10^3^21/1.47*10^3^20 = 29.49. This suggests that the data actually provide more support for the alternative hypothesis (the model not including the interaction with Group), being 29.49 more likely to occur under the alternative hypothesis compared to the null hypothesis (model including the three-way interaction).

For error rates data, comparing the H0 with the H1 in the same way as for RT data, the Bayes Factor was found to be BF_10_ = 1.82*10^−5^/4.52*10^−7^ = 40.33. This suggests that the data actually provide more support for the alternative Hypothesis (the model not including the three-way interaction with Group), being 40.33 more likely to occur under the alternative hypothesis compared to the null hypothesis (model including the three-way interaction).

## Discussion

In Experiment 1, we investigated the effect of acute stress on flexible adjustment of attention to changing distractor-response utilities in the Temporal Flanker Task. Our results essentially replicate the findings observed by Gillich et al.^16^ regarding a PCE not only for inducer items but also for diagnostic items as well as the reduction of the congruency effect over long SOA trials, suggesting concurrent processes of strategic usage and inhibitory control of distractor information.

Acute stress was successfully induced as evidenced by the VAS with increased subjective anxiety, tension and distress, as well as elevated blood pressure, and cortisol levels. Nonetheless, we neither observed an effect of acute stress on the overall congruency effect nor on modulations of the congruency effect by PC or SOA. We further observed no association between the increase in subjective stress and the PCE. That is, Experiment 1 yielded no evidence for the notion that induction of acute stress impairs monitoring of the processing of stimulus information and adjustment of processing strategies in the service of goal-directed behavior. These findings add to a growing literature reporting effects of stress on highly particular experimental conditions assumed to bear relevance with regard to cognitive control or executive functioning^7,21,23,24,42,43^. In light of the considerable heterogeneity and qualifications of result patterns obtained in these previous studies—as described in the Introduction—the lacking influence of stress in the current study is difficult to evaluate. Particularly, inferring intact monitoring of signals indicative of task strategy-relevant changes in the stimulus environment and corresponding adjustments of attention under stress would appear premature.

## Experiment 2

To broaden our empirical basis, we aimed to extend the investigation of the behavioral effects observed in Experiment 1 by conducting a second experiment, replicating the essential aspects of Experiment 1 but also featuring various procedural changes aimed to increase chances to detect potential impact of acute stress on target and distractor processing in the Temporal Flanker Task. Of most importance, we increased the contrast between PC conditions (i.e., we applied a stronger PC manipulation) and added EEG recording to obtain a more detailed view on stress effects on processing the distractor stimulus. Furthermore, we substantially increased the sample size.

Previous research involving the analysis of event-related brain potentials yielded several findings relevant for the interpretation of distractor processing characteristics. First, sensory potentials serving as classical indices for attentional selection (i.e., N1 and P1)^44^, tend to be larger in blocks of trials associated with high PC compared to low PC^15,17,19^, (but see Jost et al., ^18^ 2019, for a replication failure), suggesting adjustment of attentional weights assigned to early processing of distractor information to the overall degree of distractor utility. Second, distractors also elicit more pronounced Lateralized Readiness Potential (LRP) in favor of the hand associated with the distractor-related response in high-PC conditions than in low-PC conditions, suggesting stronger usage of the distractor for response preparation in high-PC conditions^15,17–19^. Tracking the distractor-evoked LRP during long SOA intervals revealed additional details concerning such response activation: When the target randomly occurred after either a short SOA of 350 ms or a long SOA of 1000 ms, high-PC trials displayed a bi-phasic time course of the LRP during the long SOA interval, characterized by an initial rise, temporary deactivation back to baseline, and substantial reactivation when the end of the interval approached^18^. This pattern suggests that bottom-up elicited (but strategically amplified) response activation is not maintained after realizing that the target did not occur at the early occasion, and that preparation of the response is repeated in anticipation of the late target onset.

With regard to Easterbrook’s hypothesis, it seems worth noting that priming procedures such as the Temporal Flanker Task, which allow a psychophysiological analysis of processing nominally task-irrelevant stimulus information during several hundred milliseconds prior to target onset (and thus uncontaminated by target processing), may provide a particularly well-suited means of specifying the processing stages affected by the hypothesized stress-induced shift in cognitive control.

In light of the lacking stress effect in response times in Experiment 1, we increased the difference between PC conditions, using values of 75% and 25% instead of 75% and 50%. The PC manipulation was applied to all stimuli, that is, we dropped the distinction between inducer and diagnostic items as a separate analysis of the EEG data associated with diagnostic items did not seem feasible because of the limited number of trials. Although this alteration precluded dismissal of associative learning effects as a possible source of the PCE in behavioral performance measures, it seems unlikely that early stages of distractor processing are affected by the contingency of the perceived stimulus with the response required when it is presented as a distractor. To get a detailed view on possible effects of acute stress on attentional adjustment and response preparation we included the analyses of sensory potentials (i.e., N1 and P1) and of LRP. If there are possible adjustments of attentional weights in early processing this might be visible in early sensory potentials and not be visible in the behavioral response. Furthermore, another possible target could be the response activation, where the bi-phasic pattern of the LRP in long SOA trials might be different between control and stressed participants when following the assumption of reduced top-down control in response to acute stress.

The adjustments made to the Temporal Flanker Task procedure of this Experiment 2 is essentially a replication of the experiment reported in Jost et al.^18^, with the exception that PC changed three times during the session—mirroring the procedure of Experiment 1—instead of only a single time. Finally, in order to investigate individual differences in reactivity of the ANS in more detail, we also assessed salivary alpha-amylase as a valid and reliable biomarker for ANS activity^45^.

As Booth and Sharma^7^ observed a significant PCE selectively for high-WM span participants, we also obtained individual WM spans with a Digit-Span backwards task. WM capacity has proved to be an important factor for attentional selection as demonstrated by higher levels of interference evoked by task-irrelevant stimulus information under conditions of WM load in various tasks (for an overview, see^46^). Although we know of no study that investigated the impact of WM load on the PCE, a moderating effect is suggested by findings of Kane and Engle^47^ who observed larger interference in the Stroop task for participants featuring a lower WM span which was confined, however, to conditions in which the proportion of congruent trials was high. Moreover, Soutschek et al.^48^ observed a reduced Congruency Sequence Effect (i.e., lower interference after incongruent than after congruent trials)—which some models attribute to the same processes of conflict monitoring and attentional adjustment as the PCE (see^8^ for an overview)—in conditions of additional WM load.

Based on our previous findings, we expected to replicate not only the PCE in performance measures but also a larger amplitude of distractor-elicited sensory potentials^14,17^ and of the distractor-elicited LRP^14,17,18^ under high-PC conditions than under low-PC conditions. More importantly for the purpose of the current article, however, we also exploratively examined whether acute stress affected any of these measures, potentially modulated by other factors such as working memory capacity.

### Method Experiment 2

#### Participants

In Experiment 2, we substantially increased the sample size. Seventy-one students of the Helmut-Schmidt-University/University of the Federal Armed Forces, Hamburg Germany participated in the study. All participants had normal or corrected-to-normal vision according to self-report and received partial course credit in exchange for participating. Thirty-six males (mean age: 22.8 years, range: 19–30) and thirty-five females (mean age: 22.1, range: 19–28) participated in the study.

The study was conducted in accordance with the Declaration of Helsinki with ethics approval obtained from the institutional review board of the Helmut-Schmidt University/University of Federal Armed Forces Hamburg (HSU). All procedures were carried out with written informed consent of the participants. Recruitment of participants, exclusion criteria, menstrual cycle phase for female participants who were not using hormonal contraception, assignment of participants, time of testing, and instructions for the time prior to testing were equivalent to experiment 1.

#### Apparatus, stimuli, task and procedure

The paradigm was presented on a 24″ LCD monitor and controlled by a Fujitsu PC, Intel® Core™2 Duo Processor E8500 at 3.16 GHz, Ram: 3 GB with MATLAB R2010a and Psychtoolbox-3 software. Letters A, B, C, and D served as distractor and target stimuli. Participants responded by pressing one of four response keys on a purpose-built keyboard using their index and middle fingers of both hands. Responses were mapped in alphabetical order to the response keys from left to right.

In Experiment 2, the temporal flanker task was used again with some procedural changes. Differently to Experiment 1, a 4 × 4 mapping of the stimuli was used, with the letters A, B, C, and D as distractor and target stimuli. In the high-PC condition, the distractor was highly predictive to the upcoming congruent target (75% distractor-target combinations congruent, target ‘A’ with distractor ‘A’), whereas in the low-PC condition the distractor was not predictive to the upcoming target (e.g., target ‘A’ with equal probability for each distractor). As in Experiment 1, participants either started with a block of high-PC or low-PC.

Before the main session of the experiment, participants completed the backwards digit-span task to determine individual WM capacity. The WM task took place approximately one week before the main experiment (see Waters and Caplan^49^ for stability of working memory measures). At the day of the main experiment, participants read the information about the study and gave written consent before they were seated in an acoustically and electromagnetically shielded room. While the EEG cap was adjusted, participants completed the MDBF, VAS, the Cohen’s Perceived Stress Scale (PSS, 10-item version)^50^, NEO-FFI^35^, and the State-Trait Anxiety Inventory (STAI^51^). Subsequently, participants’ vital signs were measured (blood pressure, heart rate) using an automatic wrist blood pressure monitor (Omron RS2, the Netherlands), comparably to Experiment 1 a baseline saliva sample was obtained (for description of salivary cortisol assay and salivary alpha-amylase assays see supplement). Comparable to Experiment 1, participants performed a practice block of the temporal flanker task and were then brought to a separate room for the Trier Social Stress Test (TSST ^29^) or a non-stressful control procedure of equal duration, which included the MDBF and VAS. After the TSST or control procedure, participants were brought back to the EEG lab, where the second measurement of vital signs and the second saliva sample was obtained. Again, participants completed the MDBF and VAS, before they started with the temporal flanker task. As in experiment 1, after each block mean RTs and accuracy were displayed on the screen. After every second block, the investigator took saliva samples and blood pressure readings from the participants. After the task, the fourth MDBF and VAS were completed by the participants.

#### Data analysis

For the analysis of behavioral and EEG data, the first three trials of each block (3.13%), trials with incorrect responses (2.06%), trials following incorrect responses (1.95%) and trials with response times that did not fall in the range of 200–2500 ms (0.20%) were excluded similar to Experiment 1.

As in Experiment 1, successful induction of acute stress was assured by analyzing subjective and physiological data using an ANOVA-type non-parametric test for treatment effects with global alternatives for repeated measures data in factorial designs (nparLD package v2.1 ^38^; RStudio, v1.1.463; RStudio Team 2015) with the factors Group (stress, control) and Time. Moreover, AUCi for cortisol data and increase in alpha-amylase (time point 2 – time point 1) were correlated with individual PCE values using Spearman correlation to assess the association between behavior and stress reactivity.

ANOVAs with repeated measures on the factors Congruency (congruent, incongruent), PC (high PC, low PC), SOA (short, long) and the between subjects’ factor Group (stress, control) were conducted on RTs and error rates (supplementary Tables S10 and S11) using the ez package (v4.0-0 ^40^) in RStudio (v1.1.463; RStudio Team 2015). To control for subjective stress effects on cognitive control we calculated correlations of the increase of values (i.e., timepoint 2 − timepoint 1) derived by the MDBF (elevated mood, calmness and wakefulness) and VAS (subjective anxiety, tension and distress), with the individual PCE data in the stress group using Spearman rho.

To investigate whether WM capacity moderated the stress effect as suggested by Booth & Sharma ^7^, we divided participants into low-WM capacity participants or high WM capacity participants, by splitting the groups by the median WM capacity value. A rm ANOVA for RTs and error rates with WM span as between-subjects factor was calculated.

Three participants of the initial sample had to be excluded due to artifacts in the EEG recording leaving 68 participants for the final analysis (34 stress, 34 control).

Overall, nine participants had either one missing value in the cortisol samples, that was interpolated using either linear regression (five samples) or when more than one cortisol sample was missing, the mean of the two surrounding samples was calculated (four samples). Because of at least two consecutive missing cortisol samples that could not be interpolated, we additionally excluded five participants for the analysis of cortisol (resulting in 30 stress, 33 control). Due to missing alpha-amylase values for 11 participants, analyses on alpha-amylase data included 57 participants (26 stress, 31 control). For the analysis of the VAS, we excluded six participants due to missing values resulting in 62 participants for the analysis regarding VAS (32 stress, 30 control).

#### EEG recording and analysis

EEG was recorded from 24 Ag/AgCl electrodes inserted in an elastic cap with predefined electrode positions according to the 10–20 system. Electrodes were referenced to the nose tip and horizontal and vertical electro-oculograms were recorded using bipolar montages. Electrode AFz was used as the ground electrode. To ensure a good quality of data, impedances were kept below 10 kΩ. Signals were recorded with a 32-channel amplifier (Brain Amps, Brain Products, Munich, Germany), and were sampled at 500 Hz, filtered with a low cutoff time constant of 15 s and a high cutoff frequency of 249 Hz.

Offline analyses were performed using Brain Electrical Source Analysis software (BESA, version 7.1; MEGIS GmbH, Gräfelfing, Germany). Blink artefacts were corrected using a spatial filtering method ^52^ as implemented in BESA 7.0 (BESA GmbH, Gräfeling, Germany). The EEG data was filtered off-line (0.1–30 Hz) and epochs were created starting 100 ms before distractor onset and lasting 500 ms after target onset, resulting in 800 and 1400 ms long epochs for the short and long SOA conditions, respectively. Trials with amplitude ranges that exceeded 100 µV in any of the channels were excluded (on average 16% of the trials). ERPs were extracted by averaging epochs for each participant, electrode, and experimental conditions, separately. Data was referenced to a 100 ms interval preceding the onset of the distractor for baseline correction.

For the analysis of P1 and N1 between 100 and 200 ms, the mean amplitudes around the peak for the posterior electrodes P7, P8, O1, and O2 were calculated (i.e., mean activity from 104–136 ms and 156–188 ms, locked to the onset of the distractor, for the P1 and N1, respectively). As these analyses pertained to time windows preceding target onset we did not distinguish between congruent and incongruent trials. An ANOVA was calculated with PC and SOA as within factors and Group as a between factor.

LRPs were calculated as the differences between contralateral and ipsilateral electrodes (electrodes C3 and C4) with regard to the response hand that was associated with the distractor stimulus ^17,18^. The resulting waveforms were then averaged. Negative amplitudes represent increased activations associated with the distractor stimulus. To compare activations between PC conditions and between groups, an ANOVA was calculated with PC as within factor, and Group as between factor. For the analysis of LRP in short SOA trials, an interval from 300 to 400 ms was chosen. For the analysis of LRP in long SOA trials, however, two time windows were investigated. First, the time window between 300 and 550 ms for activation associated with a target that would have occurred if it actually were a short SOA trial. And second, the time window between 800 and 1000 ms for activation associated with a target at long SOA presentation. Again, we did not distinguish between congruent and incongruent trials.

The choice of the time windows for LRP research was guided by our previous work (i.e., ^18^). In that study the (short) SOA of the target stimuli was 50 ms longer as in the current study and the distractor was presented for a longer time period (i.e., 250 ms vs. 100 ms in the current study). We adjusted the time windows for the short SOA accordingly (i.e., start of the window at 300 ms instead of 350 ms and end of the window at offset of the short SOA target). Likewise, for analysing the long SOA, the onset of the time window of the early LRP was adjusted accordingly (300 instead of 350 ms). The time window for analysis of the late LRP was also guided by findings of Jost et al. ^18^ and the analysis window was kept identical (i.e., 800–1000 ms). In addition, the analysis parameters were confirmed using the collapsed localizer technique (cf. Luck & Gaspelin ^53^), where an average across all groups and conditions is computed to define windows without the risk of overemphasizing differences.

Analysis of post-target ERPs are added in the Supplementary material.

## Results

### Subjective and physiological data

Our analyses on the MDBF (see supplementary Table S7 for descriptive data) revealed that stress significantly reduced positive mood and calmness (Group × Time: F(1,2.78) = 8.35, *p* < 0.001, and F(1,2.76) = 12.03, *p* < 0.001, respectively, Supplementary Table S5) but not wakefulness (Group × Time: F(1,2.45) = 1.24, *p* = 0.29). Using Mann–Whitney-U-Test we observed that positive mood and calmness did not differ between the two groups at the beginning of the experiment (W = 512.5, *p* = 0.419; and W = 531, *p* = 0.564, respectively) but were decreased in the stress group compared to the control group immediately after the stress/control condition (W = 743, *p* = 0.041; and W = 843.5, *p* = 0.001, respectively).

Analysis of the VAS (see Supplementary Table S8 for descriptive data) revealed that’s stress also had a significant effect on subjective anxiety (Group × Time: F(1,2.67) = 2.77, *p* < 0.046, Supplementary Table S6) and approached significance for distress (Group × Time, F(1,2.58) = 2.68, *p* = 0.054) but did not affect tension (Group × Time, F(1,2.08) = 2.23, *p* = 0.105). Again, the groups did not differ in subjective anxiety and distress at the beginning of the experiment (W = 478.5, *p* = 0.986; and W = 544.5, *p* = 0.353, respectively), but both measures were significantly higher in the stress group as compared to the control group immediately after the stress/control condition (W = 341.5, *p* = 0.042; and W = 292.5, *p* = 0.008, respectively).

**Figure 5:**
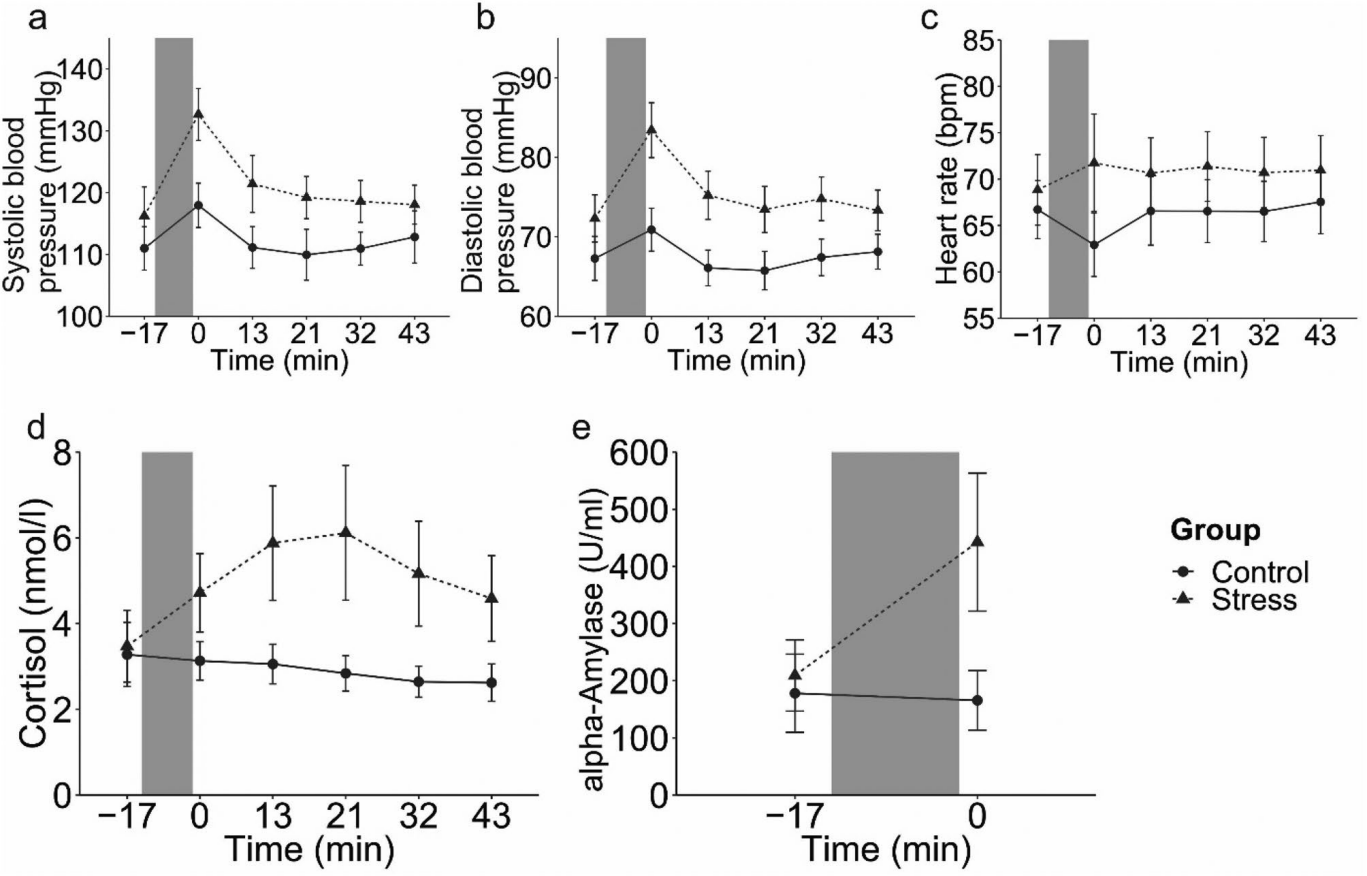
Heart rate (**a**), systolic blood pressure (**b**) diastolic blood pressure (**c**), salivary cortisol concentration (**d**) and alpha-amylase activity (**e**) for the stress and control group separately with mean data and error bars indicating 95% Confidence intervals. The gray bar indicates the stress/control manipulation.

Comparably to Experiment 1, successful stress induction was further indicated by activations of the ANS and HPA axis. Systolic and diastolic blood pressure and heart rate (Fig. 5a–c) increased in response to the TSST (Group × Time: systolic blood pressure, F(1,4.02) = 3.51, *p* = 0.007; diastolic blood pressure, F(1,4.06) = 3.68, *p* = 0.005, and heart rate, F(1,4) = 3.37, *p* = 0.009). Post-hoc Mann–Whitney-U Tests revealed that systolic blood pressure and heart rate did not differ at the beginning of the experiment (W = 454.5, *p* = 0.131; and W = 553, *p* = 0.764) were elevated in the stress group immediately after the stress condition (W = 201, *p* < 0.001; and W = 394, *p* = 0.024). Diastolic blood pressure already differed significantly between the two groups at the beginning of the experiment (W = 415, *p* = 0.046) but showed a more pronounced difference after the stress/control condition (W = 200.5, *p* < 0.001).

Finally, we observed a significant cortisol response (Fig. 5d) to the TSST (Group × Time: F(1,2.2) = 10.34, *p* < 0.001), and a significant increase in alpha-amylase activity (Fig. 5.e) in response to the TSST (F(1,4.02) = 4.6, *p* < 0.001). Post-hoc Mann–Whitney-U Tests revealed that baseline salivary cortisol concentration did not differ between control and stress group (W = 482.5, *p* = 0.869), but cortisol levels were significantly increased in the stress group until the end of the experiment (W = 327.5, *p* = 0.022; W = 260, *p* = 0.001; W = 228.5, *p* < 0.001; W = 232, *p* < 0.001; and W = 255, *p* < 0.001, for each timepoint respectively). Likewise, alpha-amylase activity did not differ significantly before the TSST or control condition (W = 326, *p* = 0.109) but showed a pronounced difference immediately thereafter (W = 189, *p* < 0.001).

### Behavioral data (Temporal Flanker Task)

**Figure 6:**
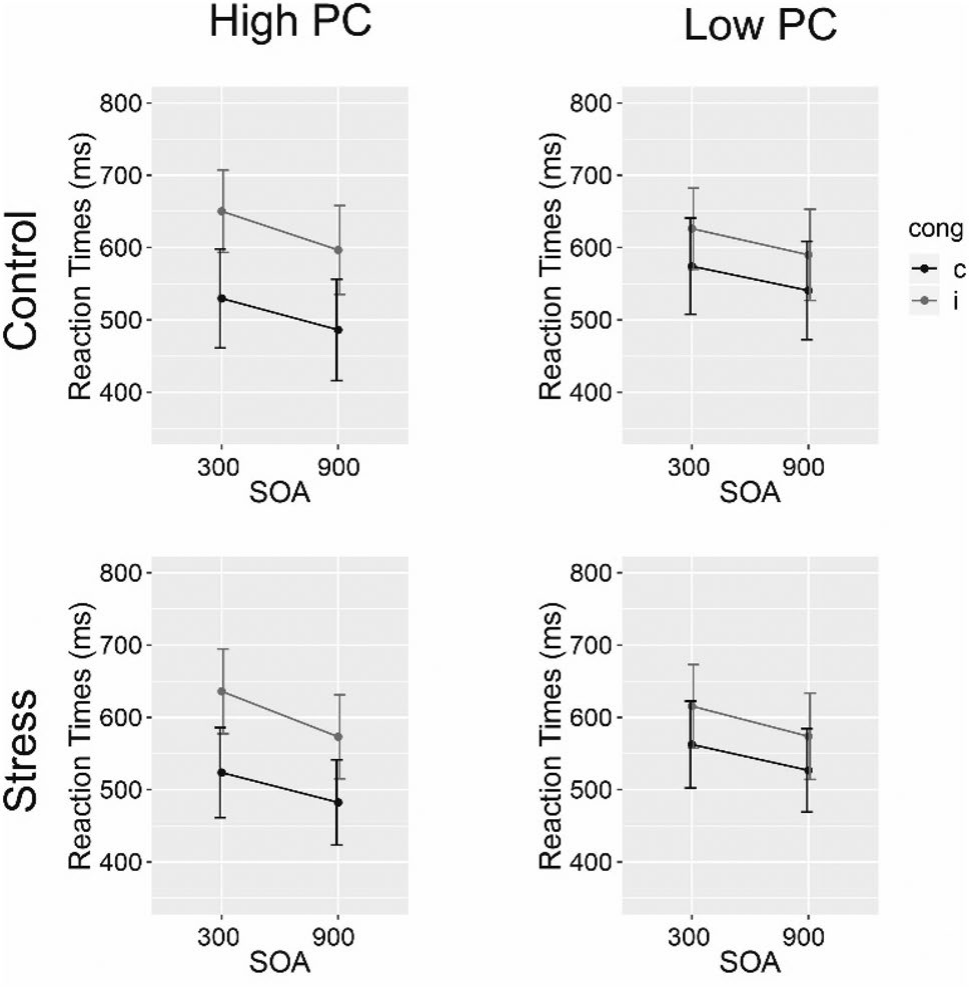
Mean RTs for the control group (top) and stress group (bottom) for congruent (c) and incongruent (i) trials in both PC conditions. Error bars indicating 95% Confidence intervals.

*Reaction times* Mean RTs are shown in Fig. 6. Similar to Experiment 1 and prior work, we observed main effects of the factors Congruency and SOA on RT, reflecting faster responses in congruent than in incongruent trials (517 ms and 605 ms, respectively) and faster responses in long SOA than in short SOA trials (539 ms and 582 ms, respectively), respectively (supplementary Table S10). Additionally, we observed a main effect of PC, reflecting faster responses in high PC compared to low PC blocks (533 ms and 589 ms, respectively). The two-way interaction PC × SOA, reached significance, reflecting a larger difference between high-PC vs. low-PC in long SOA trials (510 ms and 569 ms, for high-PC blocks and low-PC blocks, respectively) than in short SOA trials (556 ms and 608 ms, respectively). More importantly, the two-way interaction Congruency × PC reached significance, displaying a PCE (CE for high-PC blocks: 109 ms; CE for low-PC blocks: 51 ms). This two-way interaction was further modulated by SOA, reflecting that the PCE was larger for short compared to long SOA trials (64 ms and 52 ms, respectively). Most importantly for our research question and comparable to Experiment 1, we again found no effect of stress on RTs (see Fig. 6; main effect and all interactions, *p*s > 0.204, mean RT for control group: 566 ms; mean RT for stress group: 555 ms).

Furthermore, we tested whether individual PCE values in RTs were correlated with the cortisol response and the increase in alpha-amylase activity in the stress group. However, we found no evidence for an effect of cortisol release or alpha-amylase activity on attentional adjustment (rho = 0.273, *p* = 0.206 and rho = 0.088, *p* = 0.662; respectively). As in experiment 1, we also observed no correlation of the PCE with subjective anxiety, tension or distress (all rho’s < 0.18, all *p*s > 0.32).

*Error rates* The rm ANOVA of error rates (Supplementary Table S11) yielded a significant main effect of Congruency, reflecting increased error rates for incongruent trials (2.3% and 1.7%, for incongruent and congruent trials, respectively), which was modulated by PC, yielding a PCE (Ce for high-PC blocks: 1.3%; CE for low-PC blocks: 0.2%). Finally, the three-way interaction Group × PC × SOA reached significance. This effect resulted from increased error rates in the stress group for long compared to short SOA trials in low-PC blocks, whereas SOA hardly affected error rates in high-PC blocks under stress or in both high- and low-PC blocks in the control group (see Supplementary Table S9).

### Working memory capacity

When repeating the rm ANOVA for RTs and error rates with WM span as between-subjects factor we found no interactions of Group with WM-span in both data sets. We observed a three-way interaction of WM-span × Congruency × SOA in error rates (F(1,63) = 6.95, *p* = 0.011, ${\eta }_{p}^{2}$ = 0.100), reflecting increased error rates for low-span individuals in incongruent compared to congruent trials when SOA was long.

### Bayesian analysis

As in Experiment 1, our conclusions are based on null hypothesis significance testing and therefore only allow to reject the null but not the alternative hypothesis. Thus, again we repeated our analysis with a Bayesian approach with the same parameters as in experiment 1. The Bayes Factor was calculated by comparing the full model including the three-way interaction of Congruency, PC and Group (Group + Congruency + Group × Congruency + PC + Group × PC + Congruency × PC + Group × Congruency × PC) with the equivalent model excluding the three-way interaction and only including the two-way interactions of Congruency and PC, Congruency and Group, and PC and Group (Group + Congruency + Group × Congruency + PC + Group × PC + Congruency × PC).

For RT data, comparing the H0 (model including the three-way interaction of Group, Congruency, and PC) with the H1 (model without the three-way interaction) the Bayes Factor was BF_10_ = 2.41*10^6^32/2.69*10^6^31 = 8.98. This suggests that the data actually provide more support for the alternative Hypothesis (the model not including the three-way interaction), being 8.98 more likely to occur under the alternative Hypothesis compared to the null Hypothesis (model including the three-way interaction).

For error rates data, comparing the H0 with the H1 in the same way as for RT data, the Bayes Factor was found to be BF_10_ = 122.5773/1.819013 = 67.39. This suggests that the data actually provide more support for the alternative Hypothesis (the model not including the three-way interaction), being 67.39 more likely to occur under the alternative Hypothesis compared to the null hypothesis (model including the three-way interaction).

### Electrophysiological data

**Figure 7:**
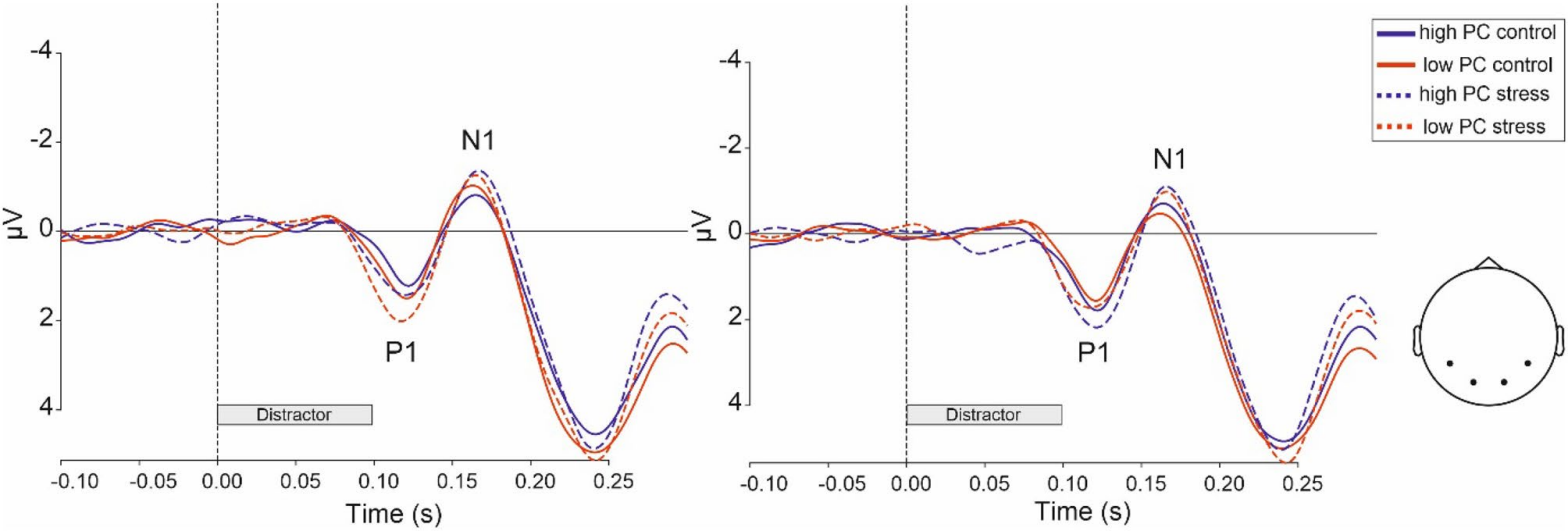
Early perceptual event-related potentials elicited by the distractor. Waveforms were averaged across four posterior electrodes (P7, P8, O1, O2) for short (left) and long (right) SOA trials.

The ANOVAs of the P1 activation elicited by the distractor (Fig. 7, between 104 and 136 ms), including the within-subject factors PC and SOA and the between-subjects factor Group yielded significant main effects of SOA (F(1,66) = 4.49, *p* = 0.038, ${\eta }_{p}^{2}$ = 0.064) () and a significant two-way interaction of PC × SOA (F(1,66) = 13.71, *p* < 0.001, ${\eta }_{p}^{2}$ = 0.172). As the SOA was chosen randomly on each trial and trials featuring a short vs. a long SOA did not differ before 300 ms after distractor onset (i.e., when the target appeared in the short SOA condition), we have no plausible explanation for the main effect of SOA and consider it to be a false-positive. Neither the main effect of Group nor the interaction of Group and PC, or Group and SOA reached significance.

In the analysis of the N1 (Fig. 7, between 156 and 188 ms), again the factor SOA reached significance (F(1,66) = 7.95, *p* = 0.01, ${\eta }_{p}^{2}$ = 0.108). Comparably to the analysis of P1 neither the main effect of Group nor the interaction of Group and PC, or Group and SOA reached significance.

**Figure 8:**
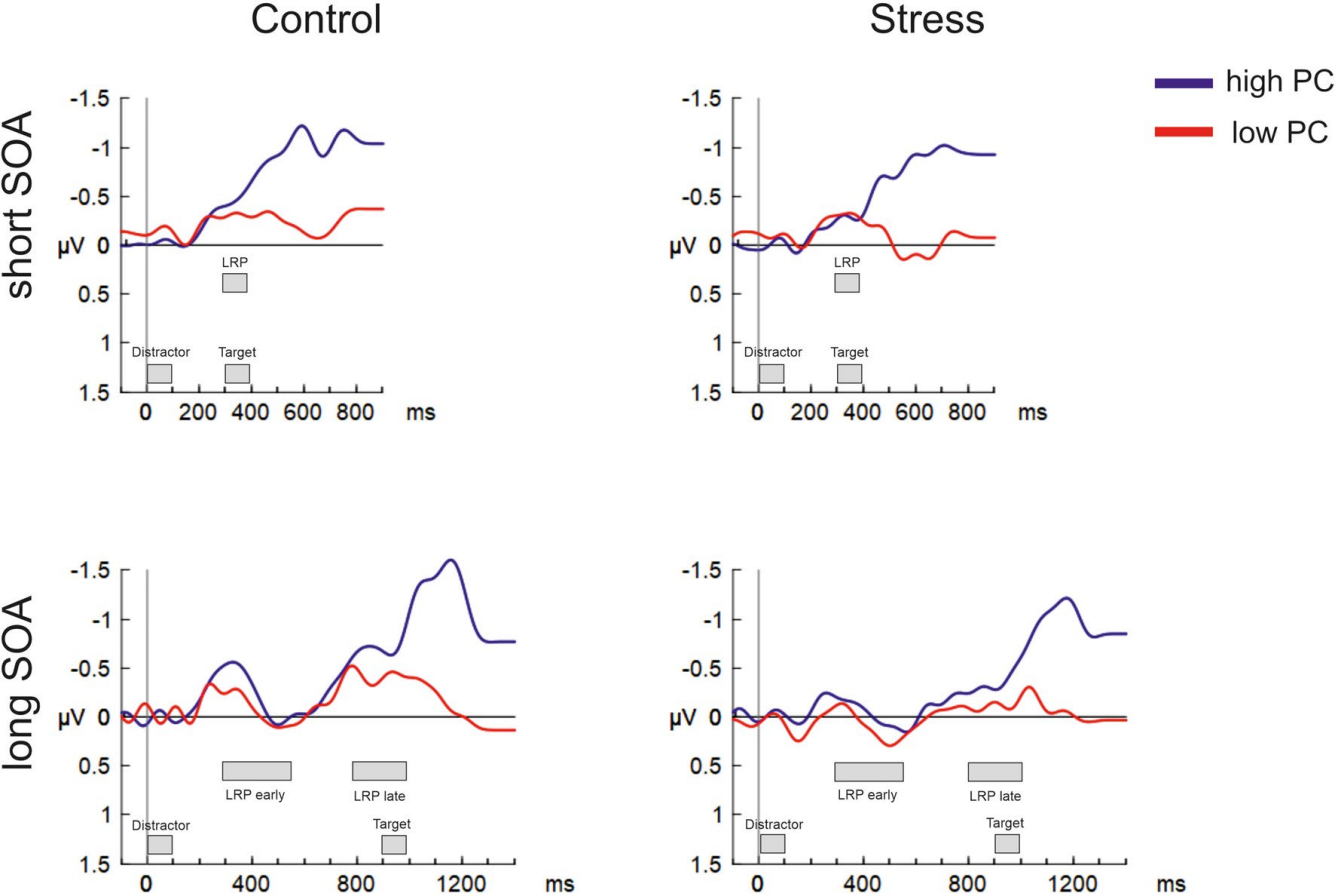
Lateralized readiness potentials time-locked to the distractor at electrodes C3/C4 for short (top) and long (bottom) SOA trials. Negative amplitudes index activations of the response hand that is associated with the distractor. LRPs were filtered with 8 Hz for this figure. Gray boxes indicate the time interval of the relative analyses.

Figure 8 displays the LRP time-locked to the distractor. Rm ANOVA for mean amplitude at short SOA trials (between 300 and 400 ms) with Group as between-subjects factor and PC as within-subject factor, revealed no main effects and no interaction effect.

Regarding long SOA trials, we analyzed mean amplitude for an early (300–550 ms) activation interval and a late (800–1000 ms) activation interval. Rm ANOVA for long SOA trials, with Group as between-subjects factor, and Time (early vs. late activation) and PC as within-subject factors, revealed main effects of Group, F(1,66) = 4.95, *p* = 0.030, ${\eta }_{p}^{2}$ = 0.070, PC, F(1,66) = 4.77, *p* = 0.033, ${\eta }_{p}^{2}$ = 0.067, and Time, F(1,66) = 10.89, *p* = 0.002, ${\eta }_{p}^{2}$ = 0.142, suggesting lower response activation in stressed participants, lower response activation in the low as compared to the high PC condition, and lower response activation during the early compared to the late response preparation time window. No interaction reached significance. Post-hoc t-tests revealed that control and stress group did not differ significantly between the high PC condition at early activation (*p* = 0.093), but were significantly different at late activation (*p* = 0.044).

## Discussion

Acute stress was again successfully induced as evidenced by decreased positive mood and calmness, increased subjective anxiety, elevated blood pressure, heart rate, cortisol levels, and additionally alpha-amylase activation. Performance data again demonstrated a PCE as well as a trend for the reduction of the congruency effect in long SOA trials.

We additionally observed a significant three-way interaction of Congruency, PC, and SOA, that was contrary to our assumption, that inhibition of distractor processing during the SOA is reduced when the distractor is assumed to be strategically used for response preparation ^16^. Surprisingly the reduction of the congruency effect was more pronounced in high-PC blocks than in low-PC blocks, or put differently, the PCE was larger when the SOA was short than when the SOA was long. Given the lack of such interaction in Experiment 1 and in the study by Gillich et al. ^16^, this effect must be considered with caution. A tentative explanation might assume that increased time of preparation for the target allows for more efficient conflict regulation which is particularly helpful in cases of strong conflict, such as in incongruent trials when PC is high.

Replicating the findings of Experiment 1, neither overall RTs, nor the congruency effect or its modulations by PC or SOA were significantly affected by the stress induction procedure. Even though stress modulated the interaction of PC and SOA in error rates, this effect was not predicted and not present in Experiment 1 and is thus difficult to interpret. Thus, the behavioral data of Experiment 2 do not extend our previous findings in providing evidence for a stress-related deficit in attentional adjustment to PC. Rather, our data accord with the notion that cognitive monitoring and adjustment processes are hardly affected by the stress induction we applied.

In line with the absence of a reduced PCE under stress in behavioral data, we furthermore observed no general stress-related reduction of sensory potentials evoked by the distractor, nor an impairment of the PC modulation of these sensory potentials. Unexpectedly, in short SOA trials the posterior P1 was more pronounced when the PC was low than when the PC was high. This finding should be considered with caution, however, as considerable differences between the conditions occurred already at an earlier stage (i.e., immediately following stimulus onset, see Fig. 7). Since we lack an explanation for these differences, we restrain ourselves from interpreting the P1 effects. It seems clear, however, that distractor related activations in the P1 and N1 components were not decreased in stressed participants, yielding no evidence for increased selective attention, and thereby decreased attention devoted to distractor stimuli after stress, that would confirm Easterbrook’s hypothesis.

The analysis of the LRP, however, provided some evidence for reduced usage of distractor information for response preparation under stress. More precisely, while in long SOA trials the distractor-elicited LRP replicated the activation-deactivation-reactivation pattern observed by Jost et al. ^18^ its amplitude was lower in the stress group than in the control group (although this effect did not reach statistical significance for the initial rise of the LRP directly following the presentation of the distractor). Our results suggest that, in contrast to control participants, stressed participants engage considerably less in response preparation on the basis of a predictive distractor, both immediately after distractor presentation and later in anticipation of the target (see Fig. 8, around 300 ms and around 900 ms). Although these findings lack correspondence in behavioral performance, reduction in distractor-elicited response activation generally fits with an interpretation of Easterbrook’s hypothesis that assumes a comparably late locus of the effect. Specifically, our data suggest that stress might not affect early perceptual processing of irrelevant stimulus information but reduce the transformation of such input into response dispositions.

## General discussion

The experiments of the current study provided novel evidence concerning the impact of stress on processing irrelevant stimuli. Analyzing several distinct components of performance and ERP data in the Temporal Flanker Task with a PC manipulation demonstrated seemingly intact processes of monitoring and adjustment under stress while at the same time revealed processing alterations regarding response preparation. The stress manipulation aside, previous critical findings could be replicated, including a PCE for a distinct set of diagnostic stimuli, deconfounded of stimulus-specific contingencies (Experiment 1), a general reduction of the congruency effect with increasing SOA (significant in Experiment 1 and displaying a trend in Experiment 2), and a more pronounced amplitude of the distractor-evoked LRP in high-PC compared to low-PC conditions. These findings corroborate extant evidence for attentional adjustment to the overall utility of distractor information, resulting in increased processing of distractor information when such processing is less detrimental to overall performance.

Performance data of both experiments neither yielded evidence for a reduced influence of distractor information on responding in a current trial (i.e., no modulation of the congruency effect) nor on the modulations of the congruency effect by PC and SOA in response to acute stress. Mechanisms of monitoring consequences of distractor processing and adjusting attention accordingly thus appeared widely unaffected by our stress manipulation. Notably, these null findings are in correspondence with Husa et al.’s ^21^ recent investigation of the effect of stress in an AX—Continuous Performance Test, which bears some similarity with the Temporal Flanker Task as the identity of a target stimulus is predicted, by varying degrees, by a preceding distractor.

Contrasting with the lack of a group difference in overt behavior, however, distractor-evoked response activation, as reflected in the LRP, was significantly attenuated under stress. These results add a new perspective to the notion of generally decreased processing of nominally task-irrelevant or distractor information under stress (i.e., Easterbrook’s hypothesis). Using successive distractor-target presentation, which provided us with the possibility to evaluate brain potentials evoked by the distractor prior to target presentation, provided more direct measures of both sensory processing and stimulus–response translation than a mere analysis of interference in response performance. Indeed, although no difference in distractor-elicited sensory potentials was observed, Experiment 2 revealed a reduction of distractor-related response activation under stress. More specifically, the long SOA interval, during which the distractor-evoked LRP could be tracked for 900 ms prior to target onset, was associated with lower LRP amplitude in stressed participants. These findings are consistent with Easterbrook’s hypothesis if it is assumed that the transformation of distractor-related information into response activation represents the locus of the effect of stress. They also correspond with Husa et al.’s ^21^ conjecture of reduced usage of proactive control under stress.

Of note, the LRP attenuation occurred during both the initial activation phase and the reactivation phase (although the former effect did not reach statistical significance on its own). While the reactivation pattern of the LRP seems to be clear evidence for strategic preparation (i.e., usage of the distractor for preparing an expected response), the initial activation may be strongly driven by a “prepared reflex” mechanism in which any stimulus sufficiently similar to a possible target would be processed, to some extent, as the target itself. Future studies assessing distractor-evoked LRPs may focus on disentangling the effects of stress on automatic/bottom-up and strategic/top-down elicitation of response activation in detail.

An open question concerns the lack of correspondence of the LRP findings with the behavioral congruency effect. Why did enhanced activation for an incorrect response (in incongruent trials) and for the correct response (in congruent trials) not result in a larger congruency effect in performance measures of the control group than in the stress group? Although it is conceivable that behavioral performance data, representing the “end product” of the entire processing chain, provide a less sensitive measure for the influence of distractor processing than online recording of brain potentials, an intriguing alternative possibility is that unstressed participants not only used distractor-based information more strongly for response preparation but also engaged more efficiently in some form of preparation for possible conflict occurrence, resulting in enhanced resolution thereof.

In summary, our findings add to a number of highly specific effects of stress observed in tasks assumed to tap on cognitive control (e.g., reduced PCE for high WM span participants ^7^; reduced accuracy in task switch trials ^24^; reduced speed of responding in task repetition trials with short preparation time ^23^; reduced amplitude of error positivity in a go/no-go task ^42^) as well as overall null findings ^21^ by demonstrating (a) widely intact processes of behavioral adaptation to the overall utility of distractor stimuli despite considerable levels of acute stress and (b) attenuated distractor-evoked response activation, both immediately after perceiving a stimulus sufficiently similar to a relevant target and later for what appears to be strategic preparation.

Viewed from a larger perspective, identifying precise mechanisms affected by stress seems difficult for various reasons, such as vague definitions of some of the theoretical key terms ^54^ and varieties of procedural differences in stress induction and task requirements. The fact that both experiments of the current study failed to demonstrate any relevant behavioral difference between the stressed groups and the control groups emphasizes another potential reason for incoherent findings, lack of sensitivity in commonly used behavioral measures. Designing experimental tasks allowing for a better interpretation of different compounds of performance measures, combined with appropriate assessment of physiological variables, seems important for further progress.

### Supplementary Information

Supplementary Information.

## Data Availability

The data that support the findings of this study are available from the corresponding author, upon reasonable request.
