# Supplementary material for: Flexible processing of distractor stimuli under stress
Source: Sci Rep. 2024 May 11;14:10824. doi: 10.1038/s41598-024-61162-8 (PMC11088623; doi:10.1038/s41598-024-61162-8)
Supplement: Supplementary file 1 — Supplementary Information. [file 41598_2024_61162_MOESM1_ESM.docx]

**Supplementary methods**

**Experiment 1**

**Description of salivary cortisol assay**

Salivary cortisol concentrations were assessed using Salivettes® Cortisol (Sarstedt, Nümbrecht), blue cap, with syntetic swabs. Participants were instructed to keep the Salivettes on one side in their cheek without chewing on them for about 90 seconds, while vital signs were measured twice. At the end of each testing day, samples were frozen and stored at -20°C. Analyses were performed using commercially available chemiluminescence immunoassays by Dresden LabService GmbH with intra- and inter-assay precision of 3.0 % and 4.2 %, respectively. Data from missing cortisol samples were estimated using linear interpolation (resulting in two estimated values from one control and one stressed participant). For two participants two consecutive samples were missing, and they were therefore excluded from analyses on cortisol data.

**Experiment 2**

**Description of salivary cortisol assay**

Salivary cortisol concentrations were again assessed using Salivettes ® Cortisol (Sarstedt, Nümbrecht), blue cap, with syntetic swabs. Participants were instructed, as in experiment 1, to keep the Salivette in their cheek for about 90 seconds while vital signs were measured twice. Samples were frozen and stored at -25 degrees C until analysis. After thawing, salivettes were centrifuged at 3,000 rpm for 5 min, which resulted in a clear supernatant of low viscosity. Salivary concentrations were measured using commercially available chemiluminescence immunoassay with high sensitivity (IBL International, Hamburg, Germany). The intra and interassay coefficients for cortisol were below 9%.

**Description of salivary alpha-amylase assays**

Concentration of alpha-amylase in saliva, collected by Salivettes ® Cortisol (Sarstedt, Nümbrecht), blue cap, with syntetic swabs, was measured by an enzyme kinetic method: Saliva was processed on a Genesis RSP8/150 liquid handling system (Tecan, Crailsheim, Germany). First, saliva was diluted 1:625 with double-distilled water by the liquid handling system. Twenty microliters of diluted saliva and standard were then transferred into standard transparent 96-well microplates (Roth, Karlsruhe, Germany). Standard was prepared from ‘‘Calibrator f.a.s.’’ solution (Roche Diagnostics, Mannheim, Germany) with concentrations of 326, 163, 81.5, 40.75, 20.38, 10.19, and 5.01 U/l alpha-amylase, respectively, and bidest water as zero standard. After that, 80 ml of substrate reagent (a-amylase EPS Sys; Roche Diagnostics, Mannheim, Germany) were pipetted into each well using a multichannel pipette. The microplate containing sample and substrate was then warmed to 37 degrees C by incubation in a waterbath for 90 s. Immediately afterward, a first interference measurement was obtained at a wavelength of 405 nm using a standard ELISA reader (Anthos Labtech HT2, Anthos, Krefeld, Germany). The plate was then incubated for another 5 min at 37 C in the waterbath, before a second measurement at 405 nm was taken. Increases in absorbance were calculated for unknowns and standards. Increases of absorbance of diluted samples were transformed to alpha-amylase concentrations using a linear regression calculated for each microplate (Graphpad Prism 4.0c for MacOSX, Graphpad Software, San Diego, CA). The intra and interassay coefficients for amylase were below 9%. (Taken from [1])

**Supplementary results**

**Experiment 1**

**Supplementary Table S1.** Subjective mood ratings from the MDBF from both experimental groups for experiment 1.

|  | Control group | | | | Stress group | | | |
| --- | --- | --- | --- | --- | --- | --- | --- | --- |
|  | Baseline | During Control condition | After Control | End of Experiment | Baseline | During TSST | After TSST | End of Experiment |
| Subjective measure |  |  |  |  |  |  |  |  |
| Bad vs. Good mood | 16.22 (3.30) | 14.04 (2.84) | 16.35 (2.96) | 16.39 (3.01) | 16.50 (2.38) | 13.33 (2.39) | 14.38 (3.73) | 15.83 (2.96) |
| Restlessness vs. Calmness | 15.22 (3.57) | 11.13 (3.55) | 15.17 (2.93) | 15.65 (3.21) | 15.67 (2.65) | 10.71 (2.44) | 12.50 (4.02) | 15.21 (3.66) |
| Sleepiness vs. Wakefulness | 14.87 (2.91) | 12.87 (2.14) | 14.48 (2.84) | 12.48 (2.69) | 15.13 (3.07) | 11.38 (2.72) | 14.25 (3.14) | 13.08 (4.56) |

Note: Data represent mean (sd).

**Supplementary Table S2.** Ratings for the VAS, consisting of three visual analogue scales to assess subjective anxiety, tension, and distress for experiment 1.

|  | Control group | | | | Stress group | | | |
| --- | --- | --- | --- | --- | --- | --- | --- | --- |
|  | Baseline | During Control condition | After Control | End of Experiment | Baseline | During TSST | After TSST | End of Experiment |
| Subjective measure |  |  |  |  |  |  |  |  |
| Subjective anxiety | 1.70 (1.11) | 2.48 (1.88) | 1.35 (0.71) | 1.26 (0.54) | 1.50 (1.14) | 4.21 (2.40) | 2.04 (1.37) | 1.17 (1.54) |
| Tension | 2.78 (1.70) | 3.78 (1.88) | 2.04 (0.98) | 1.48 (0.73) | 2.79 (1.64) | 6.21 (1.96) | 4.38 (2.30) | 1.54 (0.98) |
| Distress | 3.13 (1.94) | 3.57 (1.93) | 2.57 (1.67) | 2.65 (2.10) | 2.96 (1.88) | 5.50 (2.02) | 4.79 (2.41) | 2.33 (1.52) |

Note: Data represent mean (sd).

**Supplementary Table S3:** Mean error rates and standard deviations as a function of distractor Congruency (congruent [cong] vs. incongruent [inc] trials), Proportion Congruent during the block, and stimulus type (First two rows vs. last two rows) for the control group for experiment 1.

|  |  | High PC | | | | Low PC | | | |
| --- | --- | --- | --- | --- | --- | --- | --- | --- | --- |
|  |  | SOA | | | | | | | |
|  |  | 200 ms | | 800 ms | | 200 ms | | 800 ms | |
|  | Distractor | M | SD | M | SD | M | SD | M | SD |
| Inducer items | cong | 1.29 | 11.27 | 1.06 | 10.25 | 1.84 | 13.43 | 1.42 | 11.83 |
|  | Inc | 3.45 | 18.28 | 1.17 | 10.77 | 2.75 | 16.36 | 1.47 | 12.03 |
| Diagnostic items | cong | 4.24 | 20.17 | 1.60 | 12.55. | 3.28 | 17.82 | 2.74 | 16.34 |
|  | Inc | 4.43 | 20.60 | 1.80 | 13.31 | 3.65 | 18.78 | 2.30 | 15.02 |

**Supplementary Table S4:** Mean error rates and standard deviations as a function of distractor Congruency (congruent [cong] and vs. incongruent [inc]), Proportion Congruent during the block, and stimulus type (First two rows vs. last two rows) for the stress group for experiment 1.

|  |  | High PC | | | | Low PC | | | |
| --- | --- | --- | --- | --- | --- | --- | --- | --- | --- |
|  |  | SOA | | | | | | | |
|  |  | 200 ms | | 800 ms | | 200 ms | | 800 ms | |
|  | Distractor | M | SD | M | SD | M | SD | M | SD |
| Inducer items | cong | 1.20 | 10.88 | 1.35 | 11.55 | 2.23 | 14.76 | 1.44 | 11.90 |
|  | Inc | 2.62 | 15.98 | 1.31 | 11.40 | 1.97 | 13.90 | 1.55 | 12.37 |
| Diagnostic items | cong | 2.60 | 15.92 | 1.89 | 13.64 | 3.14 | 17.45 | 3.08 | 17.30 |
|  | Inc | 2.80 | 16.51 | 2.85 | 16.66 | 2.37 | 15.23 | 2.28 | 14.93 |

**Supplementarsy Table S5:** Results for the repeated measures ANOVA for RTs with the between factor Group (stress, control), and the within factors Congruency (congruent, incongruent), PC (high, low), Stimulus Type (inducer, diagnostic items), and SOA (short, long) in experiment 1.

| Effect | *DFn* | *DFd* | *F* | *P* | *p<.05* | $\eta_{p}^{2}$ |
| --- | --- | --- | --- | --- | --- | --- |
| Group | 1 | 45 | 2.54 | .118 |  | 0.053 |
| Congruency | 1 | 45 | 203.29 | <.001 | * | 0.819 |
| PC | 1 | 45 | 0.32 | .573 |  | 0.007 |
| Stimulus Type | 1 | 45 | 9.39 | .004 | * | 0.173 |
| SOA | 1 | 45 | 56.37 | <.001 | * | 0.556 |
| Group x Congruency | 1 | 45 | 0.07 | .787 |  | 0.002 |
| Group x PC | 1 | 45 | 2.22 | .143 |  | 0.047 |
| Group x Stimulus Type | 1 | 45 | <0.01 | .975 |  | <0.001 |
| Group x SOA | 1 | 45 | 0.64 | .428 |  | 0.014 |
| Congruency x PC | 1 | 45 | 21.61 | <.001 | * | 0.324 |
| Congruency x Stimulus Type | 1 | 45 | 2.53 | .119 |  | 0.053 |
| PC x Stimulus Type | 1 | 45 | 5.26 | .027 | * | 0.105 |
| Congruency x SOA | 1 | 45 | 17.43 | <.001 | * | 0.279 |
| PC x SOA | 1 | 45 | 5.77 | .020 | * | 0.114 |
| Stimulus Type x SOA | 1 | 45 | 3.21 | .080 |  | 0.067 |
| Group x Congruency x PC | 1 | 45 | 0.33 | .569 |  | 0.007 |
| Group x Congruency x Stimulus Type | 1 | 45 | 2.82 | .100 |  | 0.059 |
| Group x PC x Stimulus Type | 1 | 45 | 0.01 | .913 |  | <0.001 |
| Group x Congruency x SOA | 1 | 45 | 0.18 | .674 |  | 0.004 |
| Group x PC x SOA | 1 | 45 | 0.31 | .583 |  | 0.006 |
| Group x Stimulus Type x SOA | 1 | 45 | 0.39 | .537 |  | 0.009 |
| Congruency x PC x Stimulus Type | 1 | 45 | 4.11 | .049 | * | 0.084 |
| Congruency x PC x SOA | 1 | 45 | 0.82 | .371 |  | 0.018 |
| Congruency x Stimulus Type x SOA | 1 | 45 | 0.42 | .521 |  | 0.009 |
| PC x Stimulus Type x SOA | 1 | 45 | 1.10 | .300 |  | 0.024 |
| Group x Congruency x PC x Stimulus Type | 1 | 45 | 0.25 | .618 |  | 0.006 |
| Group x Congruency x PC x SOA | 1 | 45 | 0.43 | .514 |  | 0.010 |
| Group x Congruency x Stimulus Type x SOA | 1 | 45 | 0.16 | .689 |  | 0.004 |
| Group x PC x Stimulus Type x SOA | 1 | 45 | 0.10 | .758 |  | 0.002 |
| Congruency x PC x Stimulus Type x SOA | 1 | 45 | 3.72 | .060 |  | 0.076 |
| Group x Congruency x PC x Stimulus Type x SOA | 1 | 45 | 0.06 | .816 |  | 0.001 |

**Supplementary Table S6:** Results for the repeated measures ANOVA for error rates with the between factor Group (stress, control), and the within factors Congruency (congruent, incongruent), PC (high, low), Stimulus Type (inducer, diagnostic items), and SOA (short, long) in experiment 1.

| Effect | *DFn* | *DFd* | *F* | *P* | *p<.05* | $\eta_{p}^{2}$ |
| --- | --- | --- | --- | --- | --- | --- |
| Group | 1 | 45 | 0.21 | .650 |  | 0.005 |
| Congruency | 1 | 45 | 0.94 | .337 |  | 0.021 |
| PC | 1 | 45 | 0.29 | .593 |  | 0.006 |
| Stimulus Type | 1 | 45 | 8.37 | .006 | * | 0.157 |
| SOA | 1 | 45 | 8.82 | .005 | * | 0.164 |
| Group x Congruency | 1 | 45 | 0.18 | .676 |  | 0.004 |
| Group x PC | 1 | 45 | 0.03 | .870 |  | 0.001 |
| Group x Stimulus Type | 1 | 45 | 0.20 | .657 |  | 0.004 |
| Group x SOA | 1 | 45 | 3.28 | .077 |  | 0.068 |
| Congruency x PC | 1 | 45 | 4.59 | .038 | * | 0.093 |
| Congruency x Stimulus Type | 1 | 45 | 1.86 | .180 |  | 0.040 |
| PC x Stimulus Type | 1 | 45 | 0.01 | .904 |  | <0.001 |
| Congruency x SOA | 1 | 45 | 1.11 | .297 |  | 0.024 |
| PC x SOA | 1 | 45 | 2.13 | .151 |  | 0.045 |
| Stimulus Type x SOA | 1 | 45 | 0.05 | .829 |  | 0.001 |
| Group x Congruency x PC | 1 | 45 | 0.68 | .414 |  | 0.015 |
| Group x Congruency x Stimulus Type | 1 | 45 | 0.29 | .591 |  | 0.006 |
| Group x PC x Stimulus Type | 1 | 45 | <0.01 | .978 |  | <0.001 |
| Group x Congruency x SOA | 1 | 45 | 1.04 | .314 |  | 0.023 |
| Group x PC x SOA | 1 | 45 | 1.25 | .270 |  | 0.027 |
| Group x Stimulus Type x SOA | 1 | 45 | 1.65 | .206 |  | 0.035 |
| Congruency x PC x Stimulus Type | 1 | 45 | 0.02 | .895 |  | <0.001 |
| Congruency x PC x SOA | 1 | 45 | 0.24 | .628 |  | 0.005 |
| Congruency x Stimulus Type x SOA | 1 | 45 | 1.84 | .181 |  | 0.039 |
| PC x Stimulus Type x SOA | 1 | 45 | 1.17 | .286 |  | 0.025 |
| Group x Congruency x PC x Stimulus Type | 1 | 45 | 0.37 | .545 |  | 0.008 |
| Group x Congruency x PC x SOA | 1 | 45 | 0.05 | .818 |  | 0.001 |
| Group x Congruency x Stimulus Type x SOA | 1 | 45 | <0.01 | .978 |  | <0.001 |
| Group x PC x Stimulus Type x SOA | 1 | 45 | 0.43 | .514 |  | 0.010 |
| Congruency x PC x Stimulus Type x SOA | 1 | 45 | 2.19 | .146 |  | 0.046 |
| Group x Congruency x PC x Stimulus Type x SOA | 1 | 45 | <0.01 | .947 |  | <0.001 |

**Experiment 2**

**Supplementary Table S7.** Subjective mood ratings from the MDBF from both experimental groups for experiment 2.

|  | Control group | | | | Stress group | | | |
| --- | --- | --- | --- | --- | --- | --- | --- | --- |
|  | Baseline | During Control condition | After Control | End of Experiment | Baseline | During TSST | After TSST | End of Experiment |
| Subjective measure |  |  |  |  |  |  |  |  |
| Bad vs. good mood | 16.91 (2.29) | 16.24 (2.49) | 17.18 (2.11) | 16.91 (2.40) | 17.26 (2.30) | 15.06 (2.41) | 15.50 (2.25) | 16.68 (2.13) |
| Restlessness vs. Calmness | 16.47 (2.54) | 15.53 (3.14) | 17.03 (2.38) | 16.85 (2.71) | 16.85 (2.20) | 13.03 (2.77) | 14.82 (2.47) | 16.41 (2.15) |
| Sleepiness vs. Wakefulness | 13.85 (3.29) | 14.09 (3.23) | 14.26 (3.17) | 13.00 (3.76) | 14.47 (3.14) | 14.18 (3.13) | 14.12 (3.43) | 12.56 (3.33) |

Note: Data represent mean (sd).

**Supplementary Table S8.** Ratings for the VAS, consisting of three visual analogue scales to assess subjective anxiety, tension, and distress for experiment 2.

|  | Control group | | | | Stress group | | | |
| --- | --- | --- | --- | --- | --- | --- | --- | --- |
|  | Baseline | During Control condition | After Control | End of Experiment | Baseline | During TSST | After TSST | End of Experiment |
| Subjective measure |  |  |  |  |  |  |  |  |
| Subjective anxiety | 1.5 (0.86) | 2.47(1.48) | 2.8 (1.83) | 1.93 (1.28) | 1.41 (0.61) | 2.59 (1.46) | 2.38 (1.52) | 2.63 (1.58) |
| Tension | 2. 40 (1.58) | 1.33 (0.88) | 1.53 (0.90) | 2.43 (1.77) | 2.94 (1.46) | 1.53 (0.80) | 1.91 (0.93) | 2.06 (1.01) |
| Distress | 3.00 (1.78) | 2.77 (1.83) | 1.67 (1.12) | 2.20 (1.58) | 4.53 (1.95) | 4.16 (2.05) | 1.94 (0.98) | 2.81 (1.45 ) |

Note: Data represent mean (sd).

**Supplementary Table S9:** Mean error rates and standard deviations as a function of distractor Congruency (congruent [cong] vs. incongruent [inc]), and Proportion Congruent during the block.

|  |  | High PC | | | | Low PC | | | |
| --- | --- | --- | --- | --- | --- | --- | --- | --- | --- |
|  |  | SOA | | | | | | | |
|  |  | 200 ms | | 800 ms | | 200 ms | | 800 ms | |
|  | Distractor | M | SD | M | SD | M | SD | M | SD |
| Control | cong | 1.47 | 12.04 | 1.35 | 11.56 | 2.32 | 15.06 | 2.38 | 15.24 |
|  | Inc | 2.24 | 14.79 | 3.53 | 18.46 | 2.38 | 15.23 | 2.37 | 15.20 |
| Stress | cong | 1.40 | 11.74 | 1.47 | 12.02 | 1.83 | 13.42 | 2.77 | 16.41 |
|  | Inc | 2.67 | 16.14 | 2.67 | 16.12 | 1.59 | 12.51 | 2.29 | 14.96 |

**Supplementary Table S10:** Results for the repeated measures ANOVA for RTs with the between factor Group (stress, control), and the within factors Congruency (congruent, incongruent), PC (high, low), and SOA (short, long) in experiment 2.

| Effect | *DFn* | *DFd* | *F* | *P* | *p<.05* | $\eta_{p}^{2}$ |
| --- | --- | --- | --- | --- | --- | --- |
| Group | 1 | 66 | 0.36 | .550 |  | 0.005 |
| Congruency | 1 | 66 | 308.44 | <.001 | * | 0.824 |
| PC | 1 | 66 | 21.25 | <.001 | * | 0.244 |
| SOA | 1 | 66 | 175.79 | <.001 | * | 0.727 |
| Group x Congruency | 1 | 66 | 0.70 | .411 |  | 0.010 |
| Group x PC | 1 | 66 | 0.12 | .735 |  | 0.002 |
| Group x SOA | 1 | 66 | 0.10 | .756 |  | 0.001 |
| Congruency x PC | 1 | 66 | 189.30 | <.001 | * | 0.741 |
| Congruency x SOA | 1 | 66 | 3.42 | .069 |  | 0.049 |
| PC x SOA | 1 | 66 | 5.55 | .021 | * | 0.078 |
| Group x Congruency x PC | 1 | 66 | 1.64 | .204 |  | .024 |
| Group x Congruency x SOA | 1 | 66 | 0.52 | .473 |  | 0.008 |
| Group x PC x SOA | 1 | 66 | <0.01 | .967 |  | <0.001 |
| Congruency x PC x SOA | 1 | 66 | 4.85 | .031 | * | 0.068 |
| Group x Congruency x PC x SOA | 1 | 66 | 0.44 | .512 |  | 0.007 |

**Supplementary Table S11:** Results for the repeated measures ANOVA for error rates with the between factor Group, and the within factors Congruency, PC, and SOA in experiment 2.

| Effect | *DFn* | *DFd* | *F* | *P* | *p<.05* | $\eta_{p}^{2}$ |
| --- | --- | --- | --- | --- | --- | --- |
| Group | 1 | 66 | 0.30 | .584 |  | 0.005 |
| Congruency | 1 | 66 | 8.45 | .005 | * | 0.113 |
| PC | 1 | 66 | 0.80 | .375 |  | 0.012 |
| SOA | 1 | 66 | 3.23 | .077 |  | 0.047 |
| Group x Congruency | 1 | 66 | 0.60 | .442 |  | 0.009 |
| Group x PC | 1 | 66 | 0.18 | .670 |  | 0.003 |
| Group x SOA | 1 | 66 | 0.08 | .783 |  | 0.001 |
| Congruency x PC | 1 | 66 | 21.49 | .<.001 | * | 0.246 |
| Congruency x SOA | 1 | 66 | 0.43 | .514 |  | 0.006 |
| PC x SOA | 1 | 66 | 0.36 | .550 |  | 0.005 |
| Group x Congruency x PC | 1 | 66 | 0.01 | .915 |  | <0.001 |
| Group x Congruency x SOA | 1 | 66 | 1.95 | .167 |  | 0.029 |
| Group x PC x SOA | 1 | 66 | 4.00 | .050 | * | 0.057 |
| Congruency x PC x SOA | 1 | 66 | 1.54 | .218 |  | 0.023 |
| Group x Congruency x PC x SOA | 1 | 66 | 1.33 | .252 |  | 0.020 |

**Analyses of post-target ERPs (N2/P3)**

**Introduction**

Following a reviewer’s suggestion, we additionally analyzed later electrophysiological responses to target presentation to explore whether stress might affect target processing. We focused on the fronto-central N2 and the P3.

Prior investigation of the fronto-central N2, a negative deflection of the event-related potential peaking around 200-300 ms after (target) stimulus onset which has consistently been found to be increased in incongruent compared to congruent conditions in Eriksen flanker tasks (e.g., [2, 3, 4]), revealed maximum amplitude in incongruent trials when PC was high ([5, 6]). Since this condition likely involves a particularly high level of distractor-evoked conflict (assumed to result from attentional defocusing) the N2 has been taken to reflect processes of conflict monitoring or reactive control counteracting distractor-evoked interference.

Additionally, the P3 component is associated with motor inhibition (e.g., [7]). An increased stop-related P3 was found by Ramauter et al. [8] in comparison to the go-related P3, which was furthermore increased by more frequent responses. The authors argued that withholding a response under conditions of more frequent responding requires stronger inhibition.

Stress effects on both sensory potentials and the fronto-central N2 have recently been investigated in a combined stop-change paradigm, in which stop trials, signaled by the occurrence of a visual cue shortly after presentation of the putative target stimulus, required not only withholding a response to the target but also selecting another response on the basis of an additional auditory stimulus (i.e., change signal, [9]). The stop signal and the change signal could either occur simultaneously or the change signal could follow the stop signal with an SOA of 300 ms, giving participants time to prepare for the impending change demand. Whereas no modulatory effect of stress on the P1 or N1 was observed, the N2 in stop/change trials decreased with increasing SOA in a control group, but not in stressed participants. These results led the authors to conclude that stress affects the processing stage of response selection (impairing preparation for flexibly responding to change requirements) rather than perceptual filtering. Providing EEG measures not only for (post-target) response selection stages but also regarding sensory-perceptual and response-related processing of distractor information, our Experiment 2 seemed liable to add novel evidence concerning these conjectures.

**Method**

For the analyses of the N2 and P3, mean amplitudes of congruent vs. incongruent high-PC trials locked to the onset of the target, were compared at electrode FCz using rm ANOVA (for the time windows between 230-290 ms, and 330-440 ms for N2 and P3 respectively).

**Results**

For the analysis of N2 (230-290 ms) time-locked to target presentation (Supplementary Figure S1) with the within-subject factors Congruency and SOA, and the between-subject factor Group, the main effect of Congruency reached significance (F(1,66) = 7.63, p = 0.01, $\eta_{p}^{2}$ = 0.014), replicating the usually found increase in incongruent relative to congruent trials. There was also a significant main effect of SOA, F(1,66) = 21.63, p < .001, $\eta_{p}^{2}$ = 0.247). Neither the main effect of Group nor any interaction with Group reached significance (ps > .67).

For the analysis of P3 (330-410 ms) time-locked to target onset, with the same factors as for the analysis of N2, we observed a main effect of SOA (F(1,66) = 50.84, p < .001, $\eta_{p}^{2}$ = 0.435) a significant two-way interaction of Congruency X SOA (F(1,66) = 16.99, p < .001, $\eta_{p}^{2}$ = 0.205) and a significant three-way interaction of Group X Congruency X SOA (F(1,66) = 6.15, p =.016, $\eta_{p}^{2}$ = 0.085) ). These interactions reflect that the P3 tended to be larger for incongruent than for congruent stimuli in long-SOA trials, whereas the reversed pattern occurred in short-SOA trials, and that both effects were more pronounced for the control group than for the stress group.

**Supplementary Figure S1.** Event-related potentials elicited by the target stimulus. N2 (230-290 ms after target onset) and P3 components (330-410 ms after target onset) for short SOA (left) and long SOA (right) trials in high PC conditions at electrode FCz.

**Discussion**

The analysis of the N2 component showed larger amplitudes in incongruent than in congruent trials, as was expected on the assumption that this component is linked to processes of conflict monitoring or (reactive) control. Although this effect was descriptively larger in the control group than in the stress group this difference failed to reach statistical significance.

Likewise, the P3 component, in long-SOA trials, appeared larger for incongruent than for congruent trials and more so for the control than for the stress group. This pattern was reversed (i.e., P3 larger for congruent than for incongruent trials and more so for the control group), however, in the short-SOA condition. Thus, although the findings obtained in long-SOA trials seem to accord well with an assumption of reduced conflict monitoring and regulation under stress, the unexpected pattern observed for short-SOA trials precludes any firm conclusions.

Our findings add to a heterogeneous set of results obtained concerning stress effects on the N2 component in go/no-go tasks (e.g., [10, 11, 12]) or change tasks [9]. Although our experiment failed to demonstrate a significant modulation of the N2 by stress, the lacking stress effect on sensory stimulus processing together with the evidence for reduced distractor-based response activation seems broadly in line with Yildiz et al.’s [9] conclusion that stress tends to affect late, response-related processing stages rather than early perceptual processing.

***References***

1. Rohleder, N., Wolf, J.M., Maldonado, E.F., & Kirschbaum, C. The psychosocial stress-induced increase in salivary alpha-amylase is independent of saliva flow rate. Psychophysiology. 43(6):645-52. https://doi.org/10.1111/j.1469-8986.2006.00457.x (2006).

2. Folstein, J.R., Van Petten, C. Influence of cognitive control and mismatch on theN2 component of the ERP: a review. Psychophysiology 45, 152–170. https://doi.org/10.1111/j.1469-8986.2007.00602.x (2008).

3. Heil, M., Osman, A., Wiegelmann, J., Rolke, B., & Hennighausen, E. N200 in the Eriksen-task: Inhibitory executive process? Journal of Psychophysiology, 14(4), 218–225. https://doi.org/10.1027/0269-8803.14.4.218 (2000).

4. Kopp, B., Rist, F., & Mattler, U. N200 in the flanker task as a neurobehavioral tool for investigating executive control. Psychophysiology, 33, 282-294. https://doi.org/10.1111/j.1469-8986.1996.tb00425.x (1996).

5. Bartholow, B.D., Riordan, M.A., Saults, J.S., & Lust, A.A. Psychophysiological evidence of response conflict and strategic control of responses in affective priming. Journal of Experimental Social Psychology, 45, 655-666. https://doi.org/10.1016/j.jesp.2009.02.015 (2009).

6. Jost, K., Wendt, M., Luna-Rodriguez, A., & Jacobsen, T. Electrophysiological correlates of proportion congruency manipulation in a temporal flanker task. Psychophysiology, 59:e14092. https://doi.org/10.1111/psyp.1409 (2022).

7. Johnstone, S.J., Dimoska, A., Smith, J.L., Barry, R.J., Pleffer, C.B., Chiswick, D., Clarke, A.R. The development of stop-signal and Go/Nogo response inhibition in children aged 7-12 years: performance and event-related potential indices. Int. J. Psychophysiol. Off. J. Int. Organ. Psychophysiol. 63, 25–38. https://doi.org/10.1016/j.ijpsycho.2006.07.001 (2007).

8. Ramauter, J. R., Kok, A., & Ridderinkhof, K. R. Effects of stop-signal probability in the stop-signal paradigm: The N2/P3 complex further validated. Brain and Cognition, 56, 234-252. https://doi.org/ 10.1016/j.bandc.2004.07.002 (2004).

9. Yildiz, A., Wolf, O.T., & Beste, C. Stress intensifies demands on response selection during action cascading processes. Psychoneuroendocrinology, 42, 178-187. https://doi.org/10.1016/j.psyneuen.2014.01.022 (2014).

10. Jiang C, Rau PP. The detrimental effect of acute stress on response inhibition when exposed to acute stress: an event-related potential analysis. Neuroreport. 27;28(14):922-928. https://doi.org/10.1097/WNR.0000000000000859 (2017).

11. Rodeback, R. E., Hedges-Muncy, A., Hunt, I. J., Carbine, K. A., Steffen, P. R., & Larson, M. J. The Association Between Experimentally Induced Stress, Performance Monitoring, and Response Inhibition: An Event-Related Potential (ERP) Analysis. Frontiers in human neuroscience, 14, 189. https://doi.org/10.3389/fnhum.2020.00189 (2020).

12. Chang J, Hu J, Li CR, & Yu R. Neural correlates of enhanced response inhibition in the aftermath of stress. Neuroimage. 204:116212. https://doi.org/10.1016/j.neuroimage.2019.116212. (2020).
